# Promotion of cell proliferation by the proto‐oncogene *DEK* enhances oral squamous cell carcinogenesis through field cancerization

**DOI:** 10.1002/cam4.1157

**Published:** 2017-08-23

**Authors:** Takayuki Nakashima, Hiroyuki Tomita, Akihiro Hirata, Kazuhisa Ishida, Kenji Hisamatsu, Yuichiro Hatano, Tomohiro Kanayama, Ayumi Niwa, Kei Noguchi, Keizo Kato, Tatsuhiko Miyazaki, Takuji Tanaka, Toshiyuki Shibata, Akira Hara

**Affiliations:** ^1^ Department of Tumor Pathology Gifu University Graduate School of Medicine 1‐1 Yanagido Gifu 501‐1194 Japan; ^2^ Department of Oral Maxillofacial Surgery Gifu University Graduate School of Medicine 1‐1 Yanagido Gifu 501‐1194 Japan; ^3^ Division of Animal Experiment Life Science Research Center Gifu University 1‐1 Yanagido Gifu 501‐1194 Japan; ^4^ Division of Pathology Gifu University Hospital 1‐1 Yanagido Gifu 501‐1194 Japan; ^5^ Department of Diagnostic Pathology (DDP) and Research Center of Diagnostic Pathology (RC‐DiP) Gifu Municipal Hospital 7‐1 Kashima‐cho Gifu 500‐8513 Japan

**Keywords:** *DEK*, field cancerization, oncogene, squamous cell carcinoma

## Abstract

Oral squamous cell carcinoma (OSCC) develops through a multistep carcinogenic process involving field cancerization. The *DEK* gene is a proto‐oncogene with functions in genetic and epigenetic modifications, and has oncogenic functions, including cellular proliferation, differentiation, and senescence. *DEK* overexpression is associated with malignancies; however, the functional roles of *DEK* overexpression are unclear. We demonstrated that *DEK*‐expressing cells were significantly increased in human dysplasia/carcinoma in situ and OSCC. Furthermore, we generated ubiquitous and squamous cell‐specific doxycycline (DOX)‐inducible *Dek* mice (*iDek* and *iDek‐e* mice respectively). Both DOX+ *iDek* and *iDek‐e* mice did not show differences in the oral mucosa compared with DOX‐ mice. In the environment exposed to carcinogen, DOX‐treated (DOX+) *iDek* mice showed field cancerization and OSCC development. Microarray analysis revealed that *DEK* overexpression was mediated by the upregulation of DNA replication‐ and cell cycle‐related genes, particularly those related to the *G*_1_/*S* transition. Tongue tumors overexpressing *DEK* showed increased proliferating cell nuclear antigen and elongator complex protein 3 expression. Our data suggest that *DEK* overexpression enhanced carcinogenesis, including field cancerization, in OSCC by stimulating the *G*_1_/*S* phase transition and promoting DNA replication, providing important insights into the potential applications of *DEK* as a target in the treatment and prevention of OSCC.

## Introduction

Head and neck cancer is the sixth most common human cancer 1 and occurs in the oral cavity in 48% of cases; 90% of these cases are oral squamous cell carcinoma (OSCC) 2. The development of OSCC is a multistep process involving progression from normal mucosa to papillary hyperplasia, dysplasia, carcinoma in situ (CIS), and invasive squamous cell carcinoma (SCC) 3. This progression of OSCC requires the accumulation of multiple genetic, epigenetic, and chromosomal alterations, which are influenced by a patient's genetic/epigenetic predisposition and by environmental influences, including tobacco, alcohol, chronic inflammation, and human papilloma virus (HPV) infection 4, 5, 6. Tobacco use and alcohol consumption are major risk factors for oral cancer 7.

Field cancerization (also known as field defects) is a term first proposed by Slaughter et al. in 1953 8. The principle of field cancerization encompasses carcinogen‐induced early genetic/epigenetic changes in the mucosa of the oral cavity, leading to the development of precancerous lesions and additional multifocal tumors, even though the epithelium exposed to the carcinogen may have an appearance similar to that of the normal mucosa 9. Several studies have shown that tobacco smoking increases the likelihood of malignant changes toward OSCC in the proliferating epithelium 10, 11. Furthermore, increased proliferation of the epithelium is observed, even after cessation of smoking 11, suggesting that smoking‐related high proliferative activity could be a potential event involved in field cancerization. Proliferating cell nuclear antigen (PCNA), minichromosome maintenance protein (MCM) family, and cell division cycle 6 (CDC6), which are associated with regulation of the cell cycle, particularly the *G*_1_/*S* phase transition, are significantly upregulated in precancerous lesions and OSCC in the human oral cavity 12, 13, 14, 15, 16, 17. A key step in the regulation of cell proliferation is the control of the initiation of DNA synthesis by the *G*_1_/*S* transition 18, 19.

The human *DEK* oncogene was first reported to be the target of a recurrent t(6;9) translocation that generates a fusion protein with the nucleoporin CAN in a subset of patients with acute myeloid leukemia (AML) 20, 21. *DEK*, a highly conserved nuclear factor, is the only member of its protein class 22 and plays an important role in various cell processes and cellular metabolic functions, such as maintenance of global heterochromatin integrity, transcriptional control, mRNA splicing, DNA replication, DNA damage repair, and susceptibility 23. While the regulation of *DEK* has not been well‐studied, E2 factor (E2F), nuclear transcription factor Y (NF‐Y), Yin Yang 1 (YY‐1), and estrogen receptor *α* are thought to directly modulate the transcription of the *DEK* gene 24, 25, 26. Furthermore, *DEK* has been proposed to be a potential target gene of the p16‐pRB‐E2F pathway 27, 28, a key regulator of the *G*_1_/*S* transition in mammalian cells 29. The regulation of *DEK* expression by E2F transcription factors provides an explanation for the finding that *DEK* expression is induced by the activity of the high‐risk HPV E7 protein. However, the target genes of *DEK* and the mechanisms through which *DEK* affects carcinogenesis are still unclear.

Owing to its frequent upregulation in various human malignancies, *DEK* is thought to have oncogenic activities 30; additionally, targeted suppression of *DEK* may represent a new strategic approach to the treatment of cancers 31. Interestingly, *Dek*‐knockout mice do not exhibit any abnormal phenotypes compared with wild‐type mice 31, suggesting that *DEK* may be an attractive drug target.

Recently, Adams et al. 32 used an HPV16 E7‐induced transgenic mouse model of OSCC and demonstrated that *Dek* was required for the growth of head and neck SCCs. Moreover, *DEK* protein was universally upregulated in both HPV‐positive and ‐negative human SCCs relative to adjacent normal tissue 32. Furthermore, *DEK* has been shown to be upregulated in tobacco chewing‐mediated OSCC 33. Thus, *DEK* is thought to be closely associated with the carcinogenesis of OSCC through multiple mediators, including HPV and tobacco. However, it is unclear whether *DEK* is an actual proto‐oncogene or oncogene in OSCC.

In this study, we generated a doxycycline (DOX)‐inducible *Dek* transgenic mouse model for controlling the timing and localization of *DEK* overexpression. Using this model, we investigated the role of *DEK* in OSCC in both humans and mice.

## Materials and Methods

### Mice

Krt14‐Cre and Rosa26‐LSL‐rtTA‐IRES‐GFP mice were obtained from The Jackson Laboratory (Bar Harbor, ME, USA). *Rosa26‐M2rtTA* and *Tet‐O‐Dek* mice were generated as described in Supplementary Methods. All experiments were performed in accordance with the Gifu University International Animal Care and Use Committee guidelines for the use of animals.

### Human samples

All human samples were obtained from patients who had undergone surgery for resection at Gifu University Hospital. All patients provided informed consent for the use of their tissues. This study was approved by the Institutional Review Board of Gifu University.

DOX treatment, 4‐nitroquinoline 1‐oxide (4NQO) carcinogen exposure, preparation of tissue samples for counting and histological analysis, immunohistochemistry (IHC), evaluation of immunohistochemical staining, RNA extraction, and real‐time reverse transcription (RT)‐PCR, PCR array, western blot analysis, and microarray were performed using standard methods, as detailed in Data S1. PCR array data and the primers used for real‐time RT‐PCR and R are listed in Tables S1 and S2 respectively.

## Results

### *DEK* expression was associated with human OSCC

**Figure 1:**
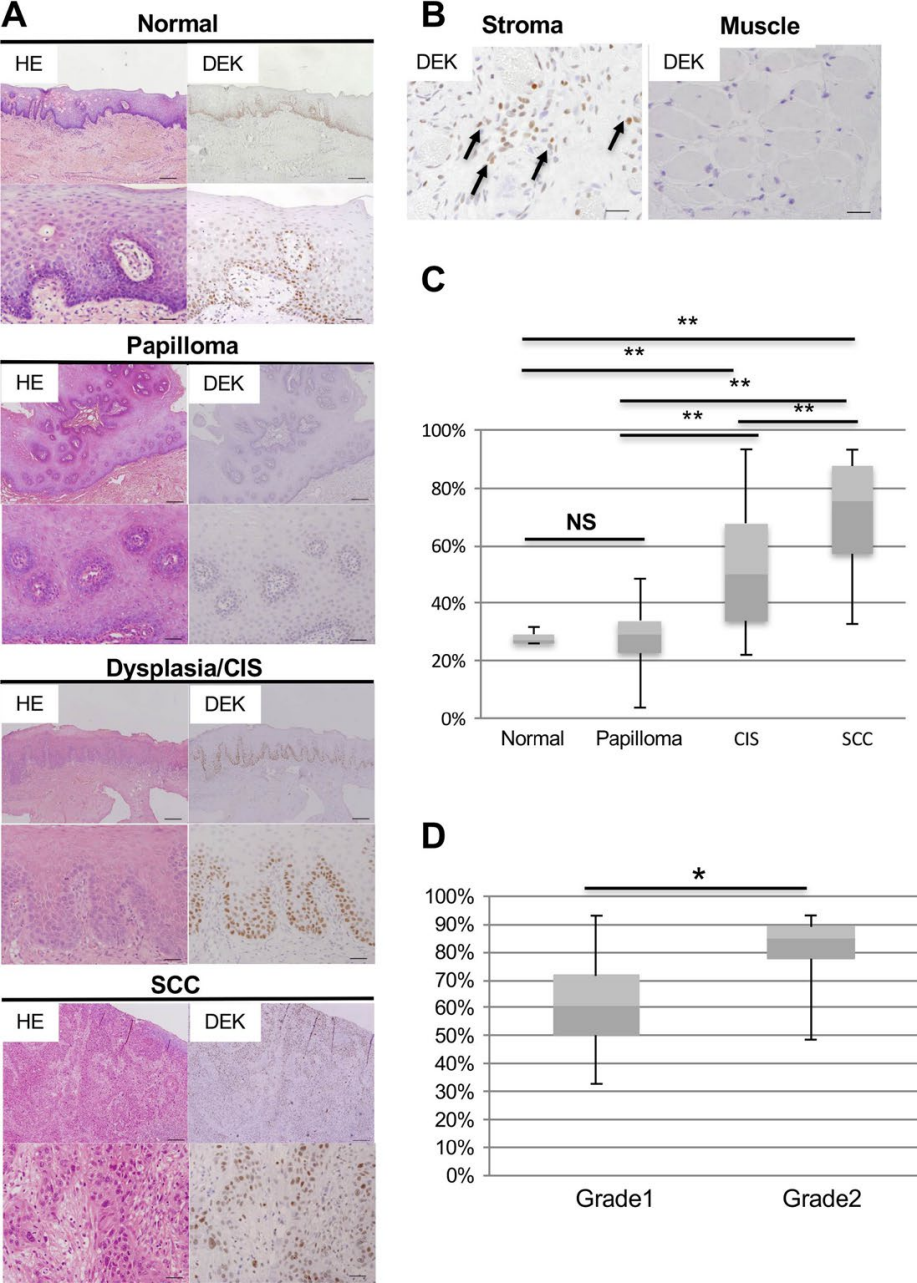
*DEK* overexpression in human OSCC. (A) Representative images of HE staining and IHC analysis of *DEK* expression in human oral tissues. Normal, normal mucosa adjacent to OSCC; CIS, carcinoma in situ; SCC, squamous cell carcinoma. Scale bars, 200 and 40 *μ*m in the upper and lower images respectively. (B) *DEK* expression in the stroma and muscle layer. Arrows indicate *DEK*‐positive cells. Scale bars, 40 *μ*m. (C) The positive index of *DEK* in normal mucosa, papilloma, CIS, and SCC in oral tissues. *Boxes:* 25th–75th percentiles; the median is the central line in each box (**P < 0.01; NS, not significant). (D) The positive index of *DEK* in grade 1 and grade 2 OSCC. Boxes: 25th–75th percentiles; the median is the central line in each box (*P < 0.05; NS, not significant).

To investigate the relationship between *DEK* expression and OSCC in humans, we performed IHC for *DEK* protein in human normal mucosa adjacent to OSCC (*n* = 5), papilloma (hyperplasia; *n* = 12), CIS (*n* = 16), and OSCC (*n* = 34). *DEK* protein predominantly showed a nuclear staining pattern with slight cytoplasmic staining in normal epithelium, papilloma, CIS, and OSCC tissues (Fig. 1A). Except in the epithelium, *DEK*‐positive cells were observed in inflammatory cells, but not muscle tissues (Fig. 1B). In the epithelium, *DEK*‐positive cells were localized at the basal layer in normal epithelium and papilloma. The *DEK*‐positive cells spread to the upper layer in CIS and were broadly extended, reaching the invasive front, in invasive SCC. The *DEK*‐positive index in OSCC was significantly higher than that of normal epithelium, papilloma, and CIS (Fig. 1C). Moreover, the *DEK*‐positive index in CIS was significantly higher than that of normal epithelium and papilloma. There were no significant differences between normal epithelium and papilloma, which are benign lesions. These results suggested that *DEK* was associated with malignant epithelial lesions in OSCC.

Next, we evaluated the *DEK*‐positive index based on histological grade (e.g., grade 1 [well‐differentiated type, *n* = 18], grade 2 [moderately differentiated type, *n* = 16], and grade 3 (poorly differentiated type, *n* = 0]), in OSCCs. The *DEK*‐positive index in grade 2 OSCC was significantly higher than that in grade 1 OSCC (Fig. 1D). These data indicated that *DEK* overexpression was closely associated with OSCC in humans.

### Inducible expression of *Dek* in ES cells and mice

**Figure 2:**
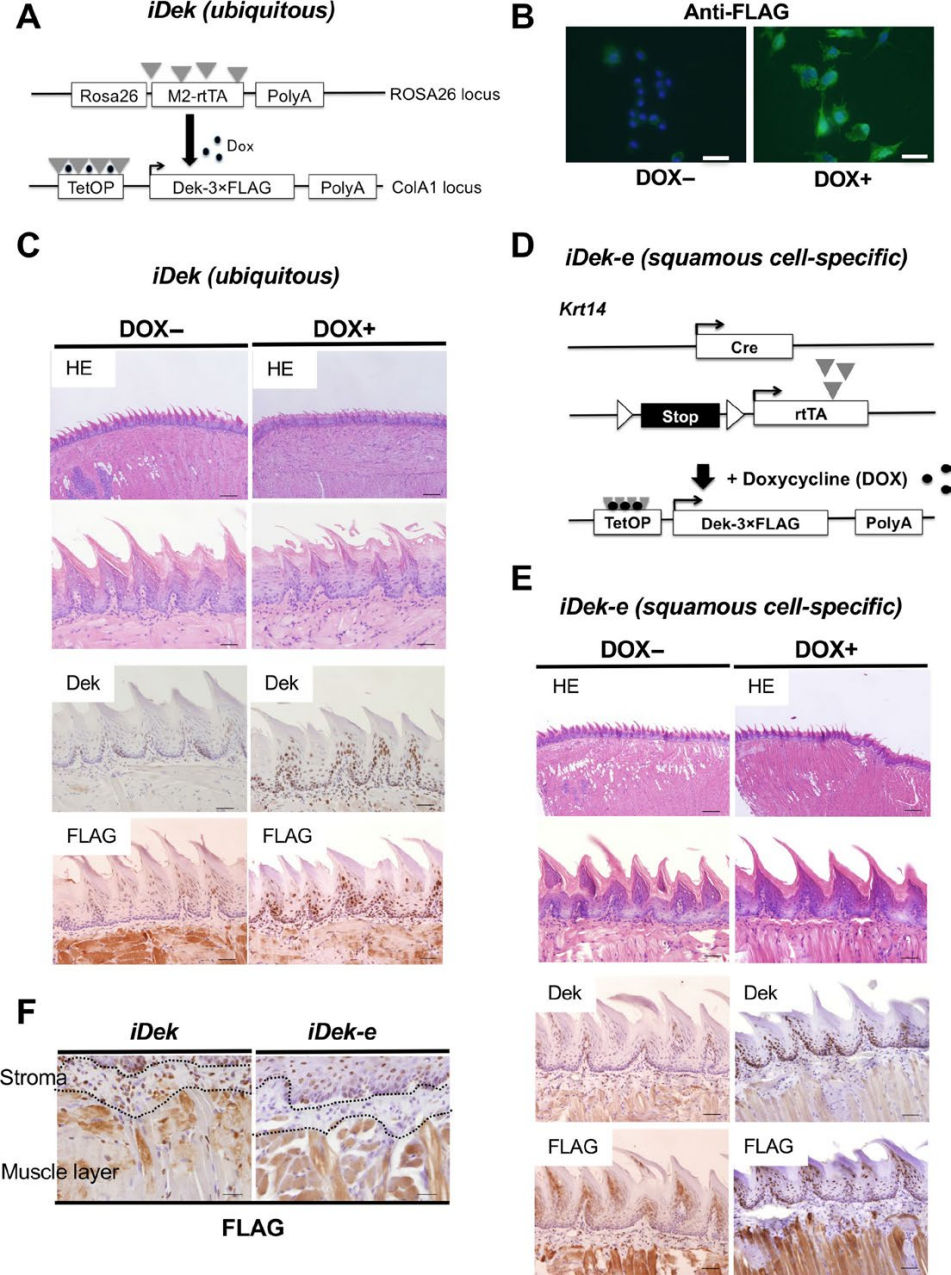
Inducible *DEK* expression in the tongues of *iDek* and *iDek‐e* mice. (A) Schematic representation of transgenes used to produce Dek‐inducible ES cells and *iDek* mice. PolyA, polyadenylation signal; TetOP, tetracycline/doxycycline‐responsive operator. (B) Immunofluorescent analysis of FLAG expression in DOX‐ and DOX+ inducible Dek ES cells after treatment with DOX (1 mg/L) for 48 h. (C) Representative images of HE and IHC staining for *DEK* and FLAG in the tongues of DOX‐ and DOX+ *iDek* mice. The concentration of DOX was 2 mg/L in the water. Scale bars, 200 and 40 *μ*m in the upper and lower images respectively. (D) Schematic representation of transgenes used to produce squamous cell epithelium‐specific *iDek* (*iDek‐e*) mice. (E) Representative images of HE staining and IHC analysis for *DEK* and FLAG expression in the tongues of DOX‐ and DOX+ *iDek* mice. Scale bars, 200 and 40 *μ*m in the upper lower images respectively. (F) FLAG expression in the stroma of tongue tissues from DOX+ *iDek* and DOX+ *iDek‐e* mice. Scale bars, 40 *μ*m.

It is unclear whether *DEK* overexpression can directly affect OSCC in vivo. Thus, to clarify the role of *DEK*, we generated a DOX‐inducible *DEK* overexpression mouse model 34, 35 to control *Dek* transcription. We first generated DOX‐inducible *Dek* ES cell clones based on the KH2 ES cell line, which harbors a modified reverse tetracycline transactivator (*M2‐rtTA*) targeted to the *ROSA26* locus (Fig. 2A). Subsequent addition of DOX to these ES cell clones resulted in upregulation of *Dek‐FLAG* (Fig. S1) and induction of the FLAG‐tagged protein (Fig. 2B). Next, *Dek*‐inducible ES cells were injected into murine blastocysts, and mice with the *TetO‐Dek* allele were obtained; heterozygous mice were used in this study.

We crossed *TetO‐Dek* mice with *Rosa26‐M2rtTA* mice to generate DOX‐inducible *Dek* expression (*iDek*) mice; *Dek* could be induced with the addition of DOX in a dose‐dependent and reversible manner (data not shown). *iDek* mice ubiquitously expressed *M2‐rtTA* driven by the murine *Rosa*26 promoter and exhibited ubiquitous *DEK* expression in vivo. To determine *DEK*‐FLAG expression in the tongue and esophagus of adult mice, we analyzed DOX‐treated (DOX+) and untreated (DOX‐) *iDek* mice for a month. There were no obvious macroscopic or microscopic changes in the tongue or esophagus between DOX+ and DOX‐ *iDek* mice (*n* = 20 each; Fig. 2C and Fig. S2A). Both *DEK* and FLAG were predominantly expressed in the basal epithelial layer of the tongue and esophagus. There were no phenotypes in the induced skin, thymus, and other tissues of *i‐Dek* mice (Fig. S3). These results suggested that forced and ubiquitous *Dek* expression in adult *iDek* mice may not contribute to visible changes under normal conditions.

Next, we generated squamous cell epithelium‐specific *iDek* (*iDek‐e*) mice by crossing *TetO‐Dek* mice with *Krt14‐Cre* and *Rosa26‐LSL‐rtTA‐IRES‐EGFP* mice (Fig. 2D). The *iDek‐e* mice overexpressed *DEK* protein only in tissues producing Krt14 protein under DOX treatment. We administered DOX to *iDek‐e* mice for 1 month and observed no obvious macroscopic or microscopic changes in the tongue and esophagus between DOX‐ and DOX+ *iDek‐e* mice (Fig. 2E, Fig. S2B). Expression of *DEK* and FLAG was observed only in the basal epithelial layer of the tongue and esophagus. Even when the observation time was extended to 1 year, there was no obvious differences in the tongue or esophagus between DOX+ *iDek* and DOX+ *iDek‐e* mice (*n* = 10 each, data not shown). These data indicated that both squamous epithelium‐specific and ubiquitous *DEK* overexpression in adult mice may not contribute to visible changes under normal conditions.

In human tissues, *DEK* was expressed in both epithelial cells and inflammatory cells (Fig. 1B). *iDek* mice broadly expressed *DEK* protein in both epithelial cells and inflammatory cells, whereas *iDek‐e* mice did not (Fig. 2F), thus indicating that *iDek* mice phenocopied human *DEK* expression whereas *iDek‐e* mice did not. Therefore, we used *iDek* mice in the carcinogenesis experiment in this study.

### *DEK* overexpression promoted the development of malignant lesions in the tongues of 4NQO‐treated mice

**Figure 3:**
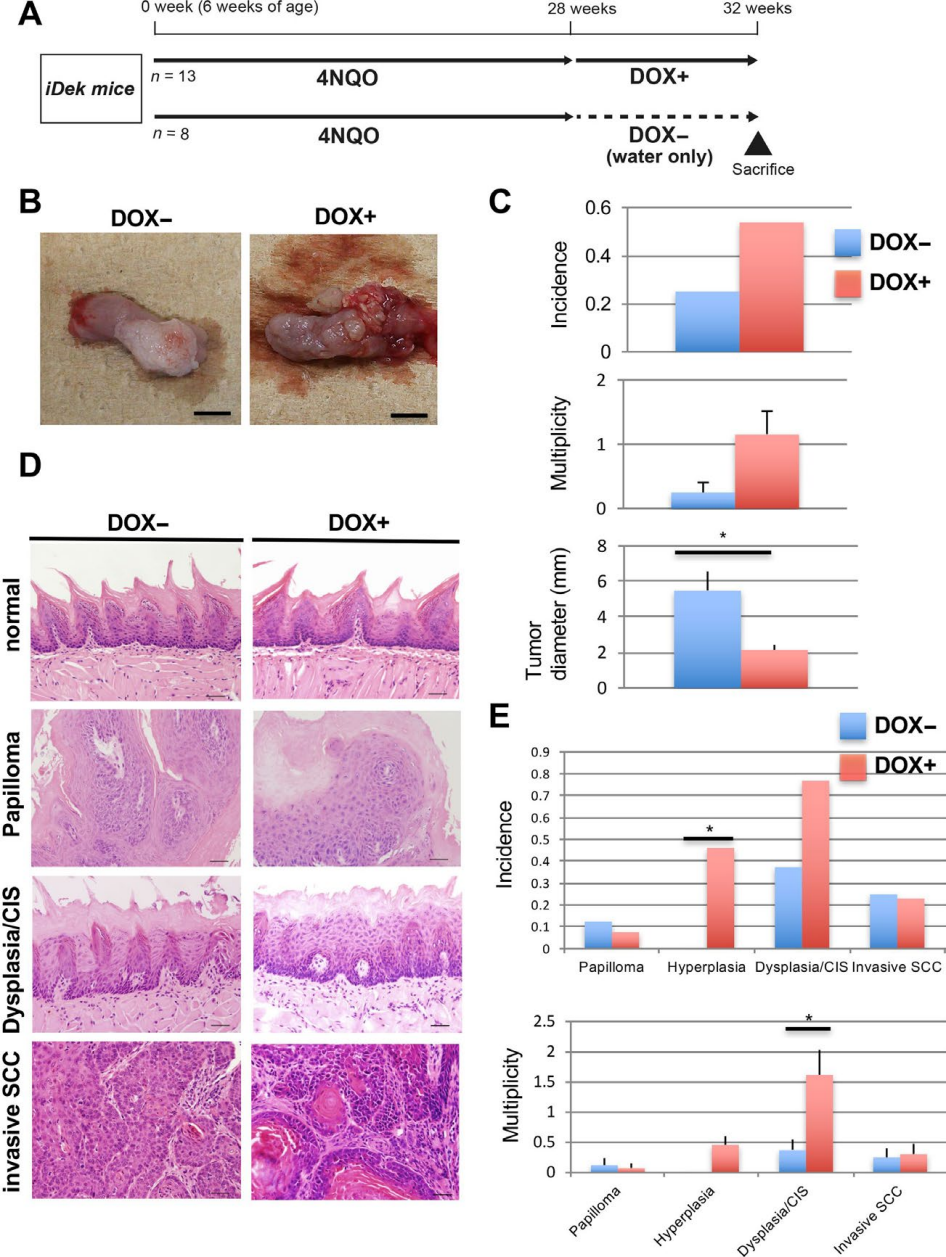
Forced *DEK* expression promoted early malignant lesions (dysplasia/CIS) in 4NQO‐treated mice. (A) Experimental protocol for this study. The concentration of 4NQO was 20 ppm. SAC, sacrifice. (B) Representative macroscopic images of tongue samples in DOX+ and DOX‐ *iDek* mice. Scale bars, 5 mm. (C) Incidence, multiplicity, and size (diameter) of macroscopic tumors of the tongue in DOX+ and DOX‐ *iDek* mice. Data are means (±SDs). ^*^ *P* < 0.05. (D) Representative microscopic images of HE staining in lesions of the tongue in DOX+ and DOX‐ *iDek* mice. Scale bars, 40 *μ*m. (E) Incidence and multiplicity of microscopic lesions of the tongue in DOX+ and DOX‐ *iDek* mice. Data are means (±SDs). ^*^ *P* < 0.05.

The 4NQO‐induced oral carcinogenesis model is very useful for elucidating the effects of carcinogens, for example, tobacco and alcohol, in human oral carcinogenesis 36 because the development of premalignant lesions and OSCC can be observed for a time even after stopping 4NQO exposure 3. To investigate whether *DEK* overexpression induced neoplastic changes in carcinogen exposure, we performed 4NQO treatment followed by DOX administration to *iDek* mice (Fig. 3A). All 4NQO‐treated mice survived during this experiment, and there were no differences in survival rates between DOX‐ and DOX+ *iDek* mice (*n* = 13 and 8 respectively). There were no detectable pathological differences in the liver, kidney, lung, or heart between DOX‐ and DOX+ *iDek* mice (data not shown).

All mice were sacrificed at 32 weeks, and tumor development was evaluated. Macroscopically nodular and polypoid tumors were found in the dorsum and lateral edge of the tongue in both cohorts (Fig. 3B). The incidences of macroscopic tongue tumors were 53% (7/13) and 25% (2/8) in DOX+ and DOX‐ *iDek* mice respectively (Fig. 3C). The multiplicities of macroscopic tongue tumors were 1.15 ± 1.29 and 0.25 ± 0.43 in DOX+ and DOX‐ *iDek* mice respectively.

In the esophagus, the incidence of macroscopic tumors was 15.4% (2/13) in DOX+ *iDek* mice; in contrast, no tumors were observed in DOX‐ *iDek* mice (0/8; Fig. S4A). There were no significant differences between DOX+ and DOX‐ mice in both the incidence and multiplicity of macroscopic tumors of the tongue. Microscopically, hyperplasia, dysplasia/CIS, and SCC were developed in the tongue and esophagus of both groups of mice (Fig. 3D and Fig. S4B). In the tongue, the multiplicity of dysplasia/CIS in DOX+ *iDek* mice was significantly higher than that in DOX‐ *iDek* mice (Fig. 3E). The incidence of hyperplasia in the tongues of DOX+ *iDek* mice was significantly higher than that in DOX‐ *iDek* mice. The incidence of dysplasia/CIS in the tongues of DOX+ *iDek* mice was obviously higher than that in DOX‐ *iDek* mice; however, this difference was not significant. *DEK* expression in dysplasia/CIS and invasive SCC were increased compared with normal and papilloma in 4NQO‐oral carcinogenesis like humans (Fig. S5 and Fig. 1). These data suggested that *DEK* overexpression was associated with the development of malignant lesions in an OSCC mouse model in the context of environmental carcinogen exposure.

### *DEK* upregulated both PCNA and elongator complex protein 3 (ELP3) during OSCC progression

*DEK* promotes cancer cell proliferation, thereby enhancing cancer growth 24. Thus, to determine the proliferation status of tumor cells in each group of mice, we performed Ki67 immunostaining in tongue lesions (Fig. S6A). There were no significant differences in Ki67 staining of tongue tissues between DOX+ *iDek* and DOX‐ *iDek* mice for tissues representing normal epithelium, dysplasia, and invasive SCC. However, the number of Ki67‐positive cells was significantly increased during tumorigenesis in each group (Fig. S6B). We analyzed the p53 positive cells in oral carcinogenesis by IHC, however, there were not significant differences during the carcinogenesis (Fig. S7A and B). These data indicated that *DEK* overexpression was unlikely to induce Ki67‐proliferating cancer cells, which are known to be present during all phases of the active cell cycle (*G*_1_, *S*,* G*_2_, and *M*), in the progression of OSCC.

**Figure 4:**
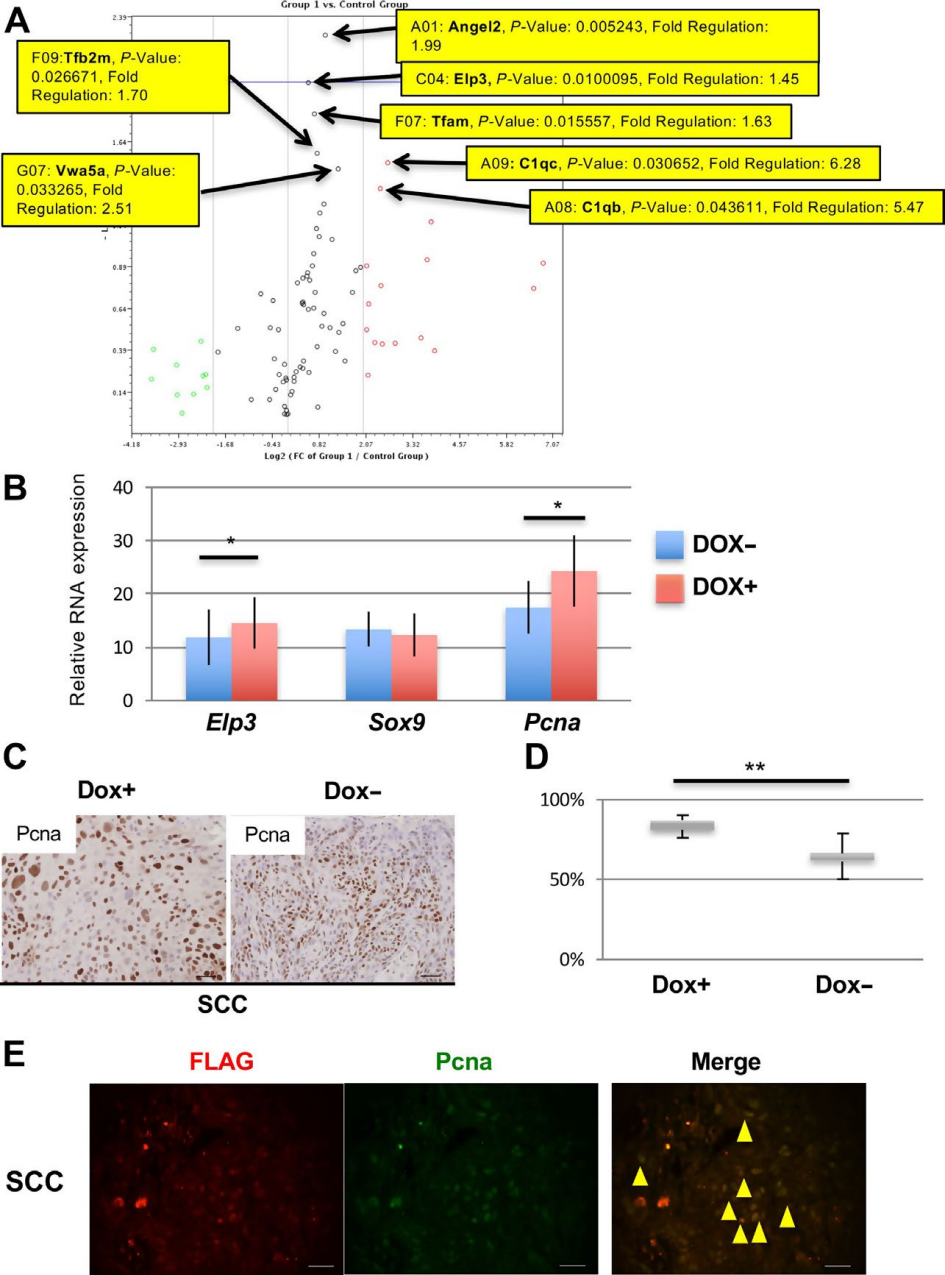
Upregulation of PCNA and ELP3 in tumor cells from DOX+ *iDek* mice treated with 4NQO. (A) Volcano plot of genes analysed using an RT2 Profiler PCR Array with a Mouse Aging set. Arrows indicate the seven genes (*P* < 0.05) with *P* values and fold changes. (B) Relative mRNA expression of *Elp3*,* Sox9*, and *Pcna* in tongue tumors of DOX‐ and DOX+ *iDek* mice (*n* = 5 each). Data are presented as the means ± SDs. ^*^ *P* < 0.05. (C) Representative images of IHC analysis of PCNA expression in tongue tumors from DOX‐ and DOX+ *iDek* mouse. Scale bars, 40 *μ*m. (D) The PCNA‐positive index in tongue tumors. Data are means ± SDs. ^*^ *P* < 0.05, ^**^ *P* < 0.01. (E) Double immunofluorescent staining with anti‐FLAG (red) and anti‐PCNA (green) antibodies in tongue tumors from DOX+ *iDek* mice treated with 4NQO. Scale bars, 20 *μ*m. Yellow arrowheads indicate representative double‐positive cells.

*DEK* is thought to act as an antagonist of senescence, and overexpression of *DEK* extends the life span of primary human keratinocytes 37. To clarify whether *DEK* overexpression contributed to the progression of malignant lesions via cellular senescence in our model, we investigated mRNA expression profiles using an RT2 Profiler PCR Array (Mouse Aging set) in tongue tumors from DOX+ *iDek* and DOX‐ *iDek* mice (*n* = 3 each). Seven (*C1qb*,* C1qc*,* Elp3*,* Tfam*,* Tfb2 m*,* Angle2*, and *Vwa5a*) of 84 aging‐related genes in the set were significantly upregulated in tumors from DOX+ *iDek* mice compared with those from DOX‐ *iDek* mice (Fig. 4A and Table S2).

ELP3 is induced by Wnt signaling and promotes SOX9 translation, which is necessary for maintenance of intestinal cancer stem cells 38, and SOX9 is upregulated in stem cell population in tongue SCC cells 39. Furthermore, ELP3 maintains genomic stability and regulates DNA replication and DNA repair by directly interacting with PCNA, which may also recruit the elongator complex to modulate chromatin structure during DNA replication or in response to DNA damage 40, 41. To confirm these findings in our model, we analysed the expression of *Sox9*,* Pcna*, and *Elp3* mRNAs in DOX+ *iDek* and DOX‐ *iDek* mice (*n* = 5 each) by real‐time RT‐RCR. *Pcna* and *Elp3* mRNA levels were significantly upregulated in tongue tumors from DOX+ *iDek* mice compared with those from DOX‐ *iDek* mice (Fig. 4B). However, PCNA, Elp3, and Sox9 protein levels were not significant differences (Fig. S8). To determine whether PCNA was functionally upregulated by *DEK* overexpression, we performed IHC for PCNA (Fig. 4C). The PCNA‐positive cells in tongue tumors of DOX+ *iDek* mice were significantly higher than those of DOX+ *iDek* mice (Fig. 4D). Furthermore, to clarify whether exogenous *DEK*‐FLAG protein induced PCNA overexpression in cancer cells, we performed double immunofluorescent staining for the FLAG tag and PCNA in tongue tumors of DOX+ *iDek* mice (Fig. 4E). Most FLAG and PCNA expression was detected in cancer cells, with obvious colocalization, in cancer cells in the tongues of DOX+ *iDek* mice treated with 4NQO, indicating that *DEK* may interact with PCNA in cancer cells during tumor progression.

### *DEK*‐dependent enhancement of field cancerization was mediated by cell cycle‐related genes, particularly during the *G*_1_/*S* transition

**Figure 5:**
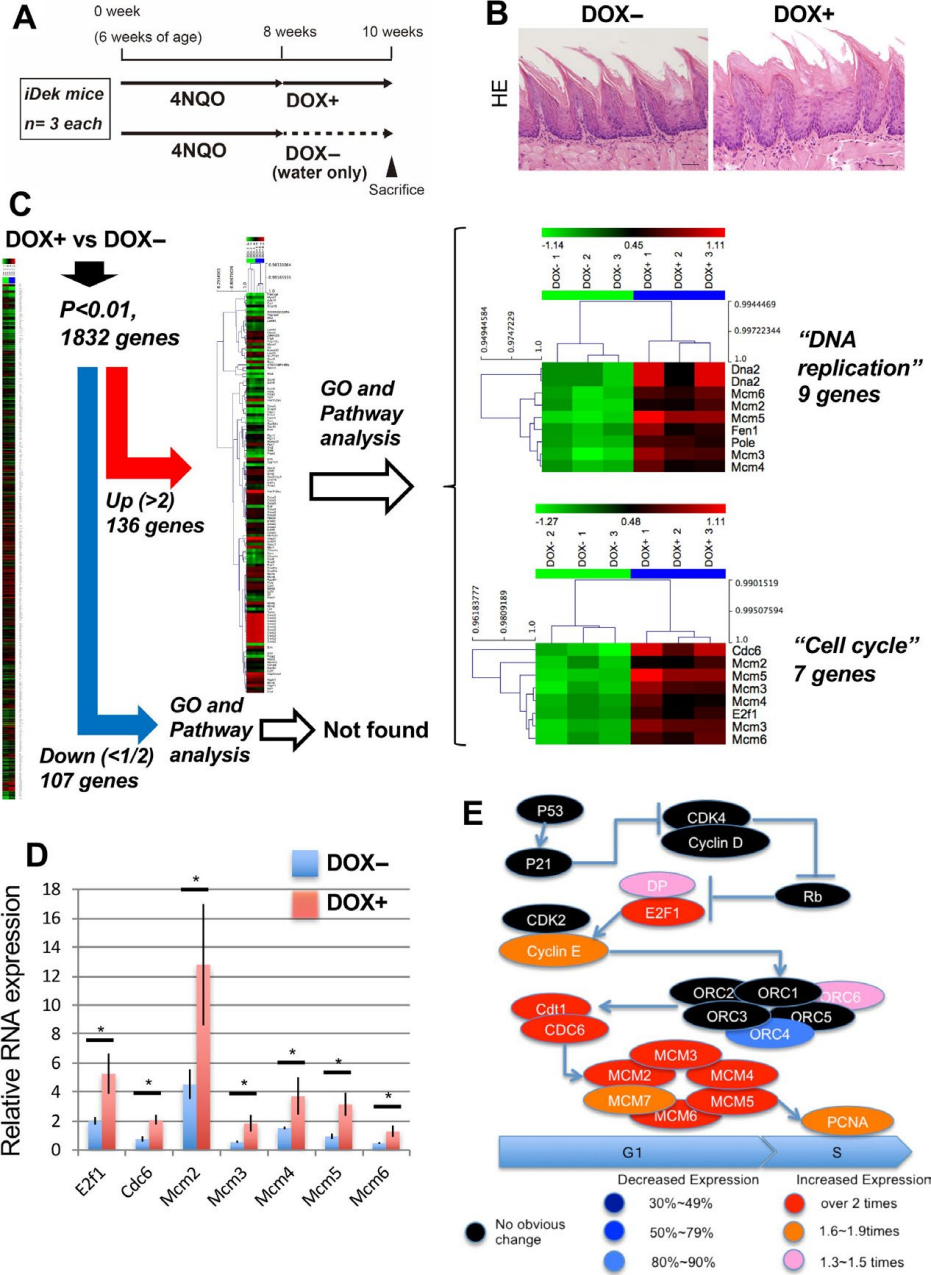
*DEK* enhanced field cancerization via upregulation of *G*_1_ check point‐related genes. (A) Experimental protocol for the short‐term experiment. (B) Representative images of HE staining of tongue tissues from 4NQO‐treated *iDek* mice followed treatment with or without DOX (DOX+ and DOX‐ respectively). Scale bars, 40 *μ*m. (C) Heat map representation of microarray analysis with GO and pathway analysis for DOX+ and DOX‐ *iDek* mice. Hierarchical clustering was performed on log2 signal intensity data. The values were resized to distance from the median for single genes (green, low expression to red, high expression). (D) RNA expression was measured by real‐time RT‐PCR for cell cycle‐associated genes. Data are means ± SDs. ^*^ *P* < 0.05. (E) The modified cell cycle pathway based on the KEGG cell cycle pathway. *G*_1_ and *S* indicate phase of the cell cycle.

PCNA‐positive cells increased during carcinogenesis. Thus, to investigate whether the occurrence of oral field cancerization was associated with carcinogen exposure in the context of *DEK* overexpression, we assessed mRNA expression profiles in the precancerous epithelium, that is, microscopically normal tissues, of the tongue in 4NQO‐treated mice with or without DOX treatment (Fig. 5A). After 10 weeks, we obtained tongue epithelial tissues showing a histologically normal appearance by HE staining (Fig. 5B) from each cohort and confirmed the absence of tumors. mRNA expression profiles were then obtained using microarray analysis. A total of 136 genes were identified as differentially upregulated (Fig. 5C). Gene ontology (GO) analysis revealed upregulation of terms associated with the cell cycle and DNA replication (seven and nine genes respectively). The seven genes belonging to the cell cycle category (i.e., *Cdc6*,* Mcm2–6*, and *E2f1*) also belonged to the cell cycle pathway in KEGG pathway analysis. Notably, for significantly downregulated genes, no significantly enriched pathways were identified. We confirmed the relative RNA expression of the upregulated genes by real‐time RT‐PCR (Fig. 5D). Interestingly, in this pathway, *Mcm7*,* cyclin E*, and *Pcna* showed a fold‐change in greater than 1.5, with a *p* value of less than 0.01 (Fig. 5E). Consequently, *DEK*‐induced cell cycle‐associated and upregulated genes were mainly related to the *G*_1_/*S* phase in the cell cycle pathway. These results indicated that *DEK* overexpression may be a risk factor promoting the precancerous environment, that is, field cancerization, in the context of carcinogen exposure by accelerating the cell cycle, particularly the *G*_1_/*S* transition, despite the apparent normal appearance of the microenvironment.

**Figure 6:**
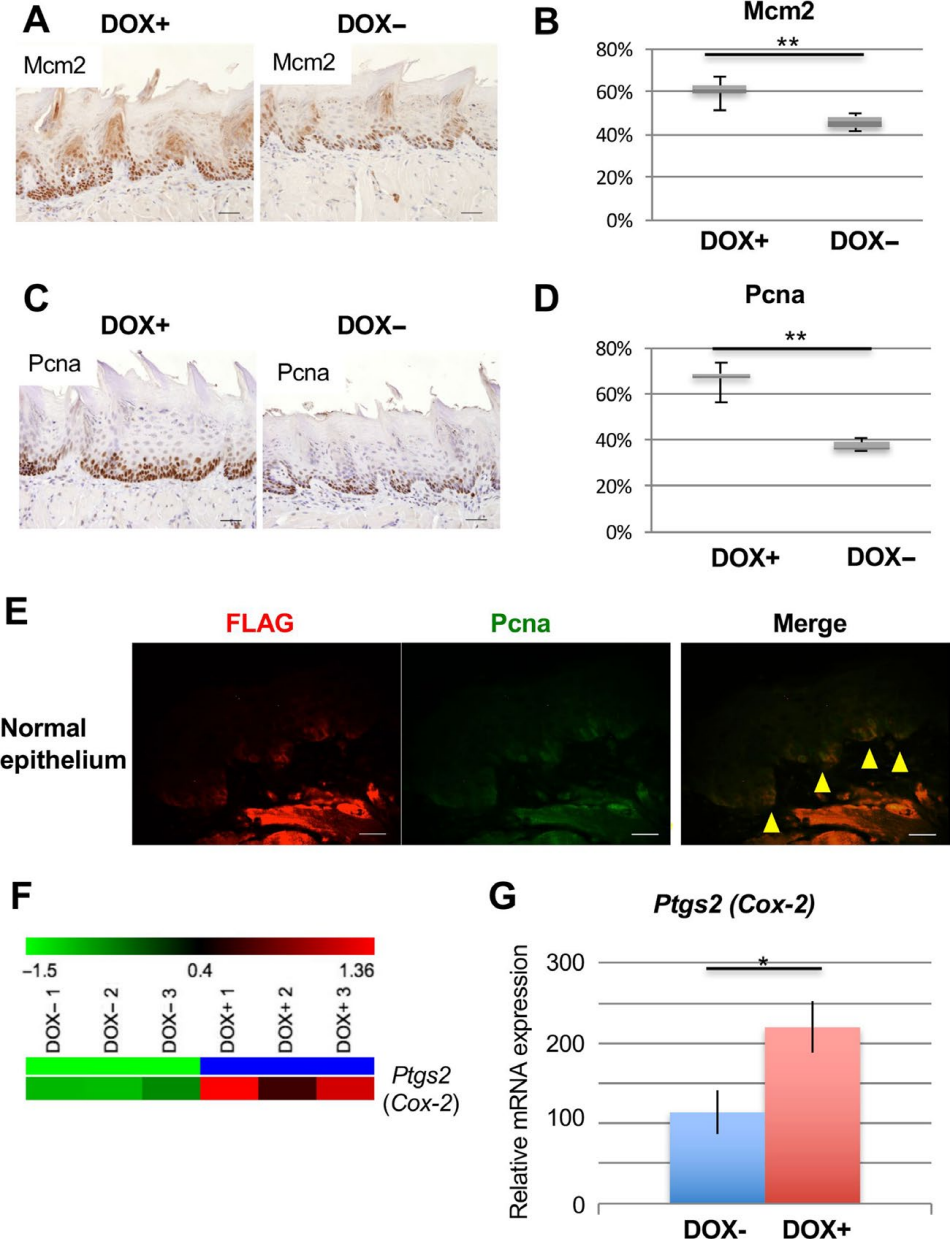
Induced *DEK* expression enhanced MCM2 and PCNA protein expression in normal‐appearing tongue epithelium exposed to 4NQO. (A) Representative images of IHC analysis for detection of MCM2 in tongue tissues from 4NQO‐treated mice with or without DOX treatment (DOX+ and DOX‐ respectively). Scale bars, 40 *μ*m. (B) The positive index of MCM2 in normal tongue epithelium from mice. Data are means ± SDs. ^**^ *P* < 0.01. (C) Representative images of IHC analysis for detection of PCNA in the tongues of 4NQO‐treated mice with or without DOX (DOX+ and DOX‐ respectively). Scale bars, 40 *μ*m. (D) The positive index of PCNA in normal tongue epithelium from mice. Data are means ± SDs. ^**^ *P* < 0.01. (E) Double immunofluorescent staining for FLAG (red) and PCNA (green) in normal tongue epithelium from DOX+ *iDek* mice. Yellow arrows indicate double‐positive epithelial cells. Scale bars, 20 *μ*m. (F) Heat map representations of microarray analysis of *Ptgs2* (*Cox‐2*) in DOX+ and DOX‐ *iDek* mice treated with 4NQO. These values were resized to the distance from the median for single genes (green, low expression to red, high expression). (G) RNA expression was measured by real‐time RT‐PCR for *Ptgs2* (*Cox‐2*). Data are means ± SDs. ^*^ *P* < 0.05.

Next, to determine whether MCM2 and PCNA protein levels increased in the normal‐appearing epithelium under carcinogen exposure, we performed IHC for MCM2 and PCNA in the tongue epithelium of DOX+ and DOX‐ mice following 4NQO treatment (using the same epithelial tissues used for the microarray analysis in the short‐term experiment; Fig. 5A). MCM2‐positive cells were localized to the basal layer of the epithelium in each cohort (Fig. 6A); however, the MCM2‐positive cells in DOX+ *iDek* mice were spread to the upper layer of the epithelium (Fig. 6A), and the MCM2‐positive index in DOX+ *iDek* mice was significantly higher than that in DOX‐ *iDek* mice (Fig. 6B). In PCNA staining, similar results were obtained (Fig. 6C and D). Furthermore, to clarify whether the exogenous *DEK*‐FLAG protein induced PCNA overexpression in squamous cells exposed to carcinogen, we performed double immunofluorescent staining for the FLAG tag and PCNA in the normal‐appearing epithelium from DOX+ *iDek* mice (Fig. 6E). Most FLAG and PCNA expression was detected in the basal layer in the tongue epithelium, with obvious colocalization, in DOX+ *iDek* mice treated with 4NQO, indicating that *DEK* may interact with PCNA in the normal‐appearing epithelium following exposure to a carcinogen.

**Figure 7:**
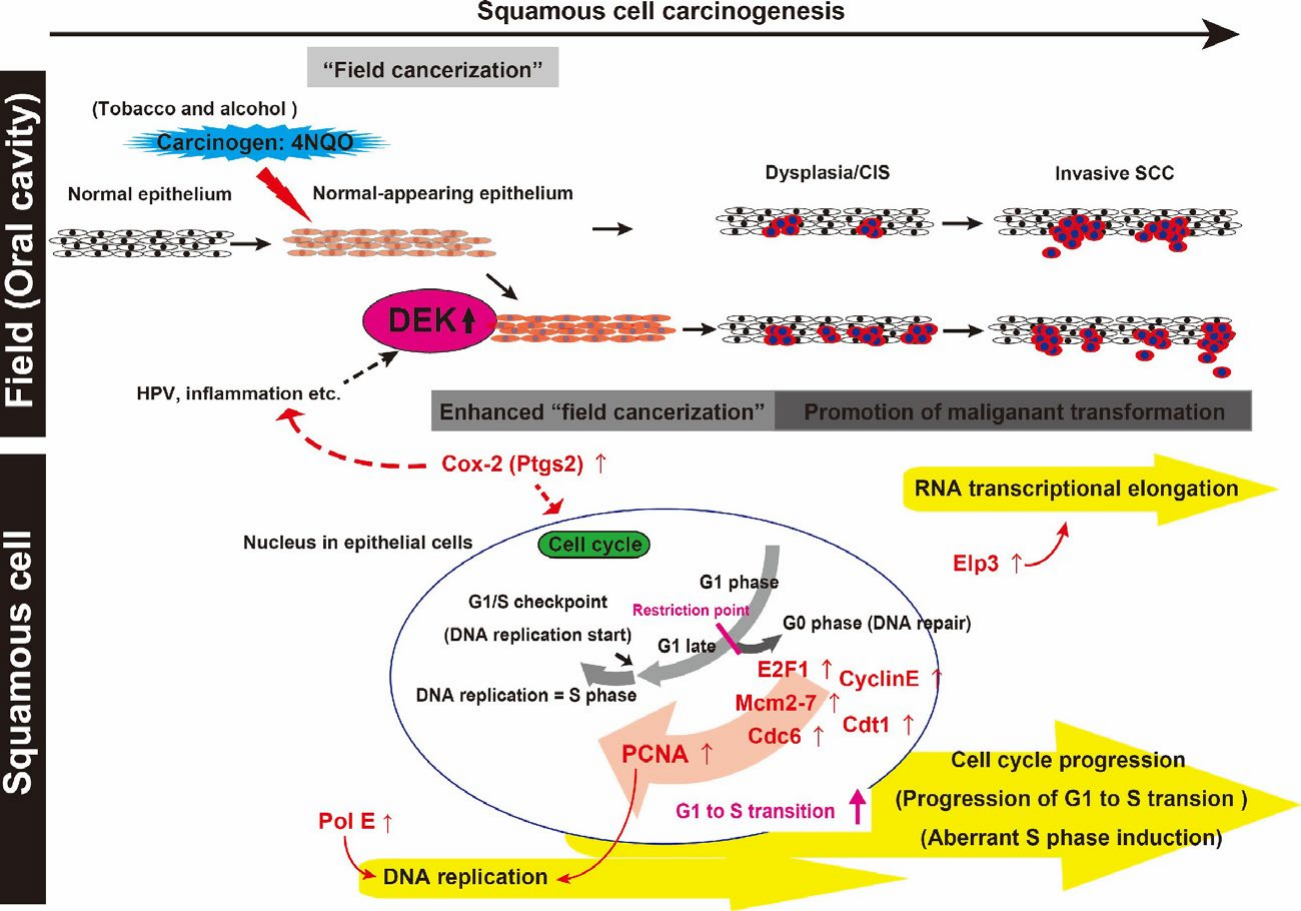
Schematic illustration of the proposed role of *DEK* in carcinogenesis of OSCC. OSCC progresses through a multistep process from normal mucosa to normal‐appearing mucosa (exposed to carcinogens, tobacco, or alcohol), hyperplasia, dysplasia, CIS, and invasive SCC. When *DEK* is upregulated and overexpressed by HPV, inflammation, or other insults, field cancerization and cancer development are promoted. Aberrant activation of the cell cycle, DNA replication, and RNA transcriptional elongation is involved in this oncogenic mechanism caused by *DEK* overexpression in OSCC. Upregulation of cell cycle progression‐, DNA replication‐, and RNA transcriptional elongation‐related genes (red) may contribute to the genomic, epigenetic, and chromosomal instabilities involved in carcinogenesis.

Among genes found to be significantly upregulated in the microarray analysis, we found that *Ptgs2* (*Cox‐2*) was upregulated in the tongues of DOX+ *iDek* mice treated with 4NQO compared with that in DOX‐ *iDek* mice treated with 4NQO in the short‐term experiment (Figs. 5A and 6F). We further confirmed *Cox‐2* upregulation by real‐time RT‐PCR (Fig. 6G). These data suggested that *Cox‐2* upregulation was also associated with the promotion of field cancerization mediated by *DEK* overexpression. *Cox‐2*, encoding cyclooxygenase 2, is known to be strongly associated with carcinogenesis in many types of cancers, including OSCC. A previous report showed that COX‐2 protein is upregulated in the normal appearing oral mucosa of tobacco smokers 42, consistent with our findings. Thus, *Cox‐2* may be related to promotion of *DEK*‐dependent field cancerization in OSCC (Fig. 7).

## Discussion

In this study, we demonstrated that *DEK* was increased in human OSCC. Moreover, in *iDek* (DOX‐inducible *Dek*) mice exposed to a carcinogen, *DEK* overexpression stimulated crucial oncogenic processes, such as DNA replication and cell cycle progression, in precancerous lesions, thereby promoting malignant transformation of tongue tumors.

*DEK* overexpression has been reported to be associated with human SCC, including the uterine cervix, head and neck, and lung 43, 44, 45, 46, 47. These studies have shown that expansion of *DEK*‐expressing cells occurs during the early phases of SCC development, e.g., in dysplasia and CIS. Our human data indicated that *DEK*‐expressing cells expanded during the early phases of SCC in the oral cavity.

We generated ubiquitous and squamous cell‐specific DOX‐inducible *Dek* mice. Ubiquitous *DEK* overexpression led to aberrant cell proliferation owing to increased DNA replication, cell cycle progression, and expression of transcriptional elongation‐related genes in epithelial tissues exposed to 4NQO, although no changes were observed in normal epithelium in either strain following DOX alone, even after 1 year. These results may be explained by the localization of cellular *DEK*. Indeed, *DEK* predominately localizes in the nucleus under steady‐state conditions; however, induction of DNA damage, apoptosis, and pro‐inflammatory factors can cause translocation of *DEK* to the extracellular space following release from inflammatory cells as a chemotactic agent 48, 49. In our study, enhancement of field cancerization by *DEK* overexpression may be related to the intracellular localization of *DEK* because there were no visible differences, e.g., inflammation, in mucosal appearance between DOX+ and DOX‐ mice following 4NQO. However, after 4NQO exposure, the number of tumors in DOX+ *iDek* mice increased compared with that in DOX‐ *iDek* mice. These findings could be related to the function of extracellular *DEK* released by inflammatory cells because many inflammatory cells are located in and around tumor tissues. Unfortunately, we were not able to distinguish intracellular and extracellular *DEK* in the tumor environment. It is necessary to consider the potential role of extracellular *DEK* in the tumor microenvironment, immunity, and/or autoimmunity and the function of both intracellular and extracellular *DEK* will be needed to be studied further in order to develop targeted therapies.

We have realized that the function of *DEK* is highly complex. Several studies have shown that *DEK* has multiple functions in DNA replication, transcriptional regulation, mRNA processing, and DNA architecture modulation 50, 51. Similarly, we found that *DEK* overexpression upregulated nine cell cycle‐related genes, particularly *G*_1_/*S* checkpoint‐related genes, and eight DNA replication‐related genes in the context of carcinogen exposure. The majority of these genes are involved in the replication‐ and transcription‐related functions of *DEK*. Furthermore, we demonstrated that ELP3, the catalytic subunit of the histone acetyltransferase elongator complex, was upregulated in tongue tumors of DOX+ *iDek* mice. ELP3, an elongator histone acetyltransferase, is required for transcriptional silencing and maintenance of genome stability and has been shown to bind directly to PCNA 41. Furthermore, the elongator complex is transported with PCNA during DNA replication to modulate chromatin structure and/or proteins involved in DNA replication to ensure that DNA synthesis is coupled to nucleosome assembly during the S phase of the cell cycle. Consequently, our data suggested that *DEK* promoted OSCC progression by mediating DNA replication and transcriptional elongation. Moreover, we showed that *DEK* could accelerate DNA replication and transcription (including RNA transcriptional elongation) in OSCC.

There were no significant differences in Ki67 staining between DOX‐ and DOX+ *iDek* mice in OSCC tissues induced by 4NQO (Fig. S5). However, PCNA‐ and MCM2‐positive cells were more abundant in the tongues of DOX+ *iDek* mice than in those of DOX‐ *iDek* mice. These findings indicated early entry of squamous cells into the *G*_1_ phase of the cell cycle. MCMs and PCNA are expressed throughout *G*_1_ phase, while Ki67 may not be expressed until late *G*_1_ phase 52. Furthermore, PCNA is a less useful marker for cell cycle entry than MCMs 53. PCNA shows maximum expression during the *S* phase, with weak staining observed in *G*_1_, *G*_2_, and *M* phases 54. PCNA is involved in the excision and replacement of abnormal nucleotides and is thus also expressed in nonproliferating cells undergoing DNA repair. This result was different from Ki67‐proliferative cells, which are all proliferating cells. Thus, PCNA‐positive proliferating cells, at least and in part, may not induce hyperplastic change in the epithelium related to *Dek* overexpression. Overall, our data clearly demonstrated that disruption and acceleration of the *G*_1_/*S* phase transition, resulting in promotion of carcinogenesis 54, occurred in OSCC. Although the functions of *DEK* have not been fully elucidated, *DEK* may have potential applications in therapeutic approaches for the prevention and treatment of OSCC.

## Conflict of Interest

The authors have no conflict of interest to disclose.

## Supporting information


**Figure S1.** *Dek*‐FLAG (exogenous) expression induced by DOX increased in three DOX‐inducible *Dek*‐FLAG KH2 ES clones.
**Figure S2.** No difference between DOX+ and DOX‐ *iDek* mice in esophagus.
**Figure S3.** No difference between DOX+ and DOX‐ *iDek* mice in skin and thymus after treatment with DOX(1 mg/L) for 2 weeks.
**Figure S4.** The high frequency of esophagus tumors in DOX+ and DOX‐ *iDek* mice.
**Figure S5.** *DEK* expression increased in the 4NQO‐ tongue carcinogenesis in both DOX+ and DOX‐*iDek* mice.
**Figure S6.** Ki67 proliferation marker increased in the tongue carcinogenesis in both DOX+ and DOX‐ *iDek* mice, but showed no significant difference between DOX+ and DOX‐ *iDek* mice.
**Figure S7.** No difference between p53 positive cells in tongue lesions of DOX+ and DOX‐ *iDek* mice.
**Figure S8.** Western blot analyses for Sox9, PCNA, and Elp3 of tumor between overexpression of *Dek* or not.

**Table S1**. PCR array data in the tongue tumors between DOX+ and DOX‐ *iDek* mice.

**Table S2.** Primers used for quantitative real‐time RT‐PCR.

**Data S1.** Details of experimental methods in this study.

## References

[1] Pai, S. I. , and W. H. Westra . 2009 Molecular pathology of head and neck cancer: implications for diagnosis, prognosis, and treatment. Annu. Rev. Pathol. 4:49–70. 18729723 10.1146/annurev.pathol.4.110807.092158 PMC3703474

[2] Jemal, A. , R. Siegel , E. Ward , Y. Hao , J. Xu , and M. J. Thun . 2009 Cancer statistics, 2009. CA Cancer J. Clin. 59:225–249. 19474385 10.3322/caac.20006

[3] Tanaka, T. , and R. Ishigamori . 2011 Understanding carcinogenesis for fighting oral cancer. J. Oncol. 2011:603740. 21772845 10.1155/2011/603740 PMC3136173

[4] Choi, S. , and J. N. Myers . 2008 Molecular pathogenesis of oral squamous cell carcinoma: implications for therapy. J. Dent. Res. 87:14–32. 18096889 10.1177/154405910808700104

[5] Gasche, J. A. , and A. Goel . 2012 Epigenetic mechanisms in oral carcinogenesis. Future Oncol. 8:1407–1425. 23148615 10.2217/fon.12.138 PMC3569850

[6] Gillison, M. L. , W. M. Koch , R. B. Capone , M. Spafford , W. H. Westra , L Wu , et al. 2000 Evidence for a causal association between human papillomavirus and a subset of head and neck cancers. J. Natl Cancer Inst. 92:709–720. 10793107 10.1093/jnci/92.9.709

[7] Blot, W. J. , J. K. McLaughlin , D. M. Winn , D. F. Austin , R. S. Greenberg , S. Preston‐Martin , et al. 1988 Smoking and drinking in relation to oral and pharyngeal cancer. Cancer Res. 48:3282–3287. 3365707

[8] Slaughter, D. P. , H. W. Southwick , and W. Smejkal . 1953 Field cancerization in oral stratified squamous epithelium; clinical implications of multicentric origin. Cancer 6:963–968. 13094644 10.1002/1097-0142(195309)6:5<963::aid-cncr2820060515>3.0.co;2-q

[9] Braakhuis, B. J. , M. P. Tabor , J. A. Kummer , C. R. Leemans , and R. H. Brakenhoff . 2003 A genetic explanation of Slaughter's concept of field cancerization: evidence and clinical implications. Cancer Res. 63:1727–1730. 12702551

[10] Shin, D. M. , N. Voravud , J. Y. Ro , J. S. Lee , W. K. Hong , and W. N. Hittelman . 1993 Sequential increases in proliferating cell nuclear antigen expression in head and neck tumorigenesis: a potential biomarker. J. Natl Cancer Inst. 85:971–978. 8098774 10.1093/jnci/85.12.971

[11] van Oijen, M. G. , M. M. Gilsing , G. Rijksen , G. J. Hordijk , and P. J. Slootweg . 1998 Increased number of proliferating cells in oral epithelium from smokers and ex‐smokers. Oral Oncol. 34:297–303. 9813726 10.1016/s1368-8375(98)00007-4

[12] Zakaria, S. H. , H. A. Farag , and D. S. Khater . 2016 Immunohistochemical expression of MCM‐2 in oral epithelial dysplasias. Appl. Immunohistochem. Mol. Morphol. 10.1097/PAI.0000000000000330 26990745

[13] Valverde, L. F. , R. D. de Freitas , T. A. Pereira , M. F. de Resende , I. M. Agra , J. N. Dos Santos , et al. 2016 MCM3: a novel proliferation marker in oral squamous cell carcinoma. Appl. Immunohistochem. Mol. Morphol. 10.1097/PAI.0000000000000397 27258565

[14] Ohnishi, Y. , T. Fujii , Y. Ugaki , et al. 2016 Usefulness of a fluorescence visualization system for the detection of oral precancerous and early cancerous lesions. Oncol. Rep. 36:514–520. 27121913 10.3892/or.2016.4776

[15] Tamura, T. , K. Shomori , T. Haruki , K. Nosaka , Y. Hamamoto , T. Shiomi , et al. 2010 Minichromosome maintenance‐7 and geminin are reliable prognostic markers in patients with oral squamous cell carcinoma: immunohistochemical study. J. Oral Pathol. Med. 39:328–334. 20136698 10.1111/j.1600-0714.2009.00861.x

[16] Li, J. N. , C. J. Feng , Y. J. Lu , H. J. Li , Z. Tu , G. Q. Liao , et al. 2008 mRNA expression of the DNA replication‐initiation proteins in epithelial dysplasia and squamous cell carcinoma of the tongue. BMC Cancer 8:395. 19116018 10.1186/1471-2407-8-395 PMC2648984

[17] Feng, C. J. , H. J. Li , J. N. Li , Y. J. Lu , and G. Q. Liao . 2008 Expression of Mcm7 and Cdc6 in oral squamous cell carcinoma and precancerous lesions. Anticancer Res. 28:3763–3769. 19189662

[18] Brand, N. , T. Faul , and F. Grummt . 2007 Interactions and subcellular distribution of DNA replication initiation proteins in eukaryotic cells. Mol. Genet. Genomics 278:623–632. 17680271 10.1007/s00438-007-0278-1

[19] Bell, S. P. , and A. Dutta . 2002 DNA replication in eukaryotic cells. Annu. Rev. Biochem. 71:333–374. 12045100 10.1146/annurev.biochem.71.110601.135425

[20] von Lindern, M. , D. Breems , S. van Baal , H. Adriaansen , and G. Grosveld , 1992 The translocation (6;9), associated with a specific subtype of acute myeloid leukemia, results in the fusion of two genes, dek and can, and the expression of a chimeric, leukemia‐specific dek‐can mRNA. Mol. Cell. Biol. 12:1687–1697. 1549122 10.1128/mcb.12.4.1687-1697.1992 PMC369612

[21] von Lindern, M. , D. Breems , S. van Baal , H. Adriaansen , and G. Grosveld . 1992 Characterization of the translocation breakpoint sequences of two DEK‐CAN fusion genes present in t(6;9) acute myeloid leukemia and a SET‐CAN fusion gene found in a case of acute undifferentiated leukemia. Genes Chromosom. Cancer 5:227–234. 1384675 10.1002/gcc.2870050309

[22] Lin, L. , J. Piao , W. Gao , Y. Piao , G. Jin , Y. Ma , et al. 2013 DEK over expression as an independent biomarker for poor prognosis in colorectal cancer. BMC Cancer 13:366. 23902796 10.1186/1471-2407-13-366 PMC3751154

[23] Piao, J. , Y. Shang , S. Liu , Y. Piao , X. Cui , Y. Li , et al. 2014 High expression of DEK predicts poor prognosis of gastric adenocarcinoma. Diagn. Pathol. 9:67. 24650035 10.1186/1746-1596-9-67 PMC3994479

[24] Privette Vinnedge, L. M. , S. M. Ho , K. A. Wikenheiser‐Brokamp , and S. I. Wells . 2012 The DEK oncogene is a target of steroid hormone receptor signaling in breast cancer. PLoS ONE 7:e46985. 23071688 10.1371/journal.pone.0046985 PMC3468546

[25] Carro, M. S. , F. M. Spiga , M. Quarto , V. Di Ninni , S. Volorio , M. Alcalay , et al. 2006 DEK expression is controlled by E2F and deregulated in diverse tumor types. Cell Cycle 5:1202–1207. 16721057 10.4161/cc.5.11.2801

[26] Sitwala, K. V. , K. Adams , and D. M. Markovitz . 2002 YY1 and NF‐Y binding sites regulate the transcriptional activity of the Dek and Dek‐can promoter. Oncogene 21:8862–8870. 12483538 10.1038/sj.onc.1206041

[27] Vernell, R. , K. Helin , and H. Muller . 2003 Identification of target genes of the p16INK4A‐pRB‐E2F pathway. J. Biol. Chem. 278:46124–46137. 12923195 10.1074/jbc.M304930200

[28] Muller, H. , A. P. Bracken , R. Vernell , M. C. Moroni , F. Christians , E. Grassilli , et al. 2001 E2Fs regulate the expression of genes involved in differentiation, development, proliferation, and apoptosis. Genes Dev. 15:267–285. 11159908 10.1101/gad.864201 PMC312619

[29] Sherr, C. J. 1996 Cancer cell cycles. Science 274:1672–1677. 8939849 10.1126/science.274.5293.1672

[30] Privette Vinnedge, L. M. , F. Kappes , N. Nassar , and S. I. Wells . 2013 Stacking the DEK: from chromatin topology to cancer stem cells. Cell Cycle 12:51–66. 23255114 10.4161/cc.23121 PMC3570517

[31] Wise‐Draper, T. M. , R. A. Mintz‐Cole , T. A. Morris , D. S. Simpson , K. A. Wikenheiser‐Brokamp , M. A. Currier , et al. 2009 Overexpression of the cellular DEK protein promotes epithelial transformation in vitro and in vivo. Cancer Res. 69:1792–1799. 19223548 10.1158/0008-5472.CAN-08-2304 PMC2650744

[32] Privette Vinnedge, L. M. , N. M. Benight , P. K. Wagh , N. A. Pease , M. A. Nashu , J. Serrano‐Lopez , et al. 2015 The DEK oncogene promotes cellular proliferation through paracrine Wnt signaling in Ron receptor‐positive breast cancers. Oncogene 34:2325–2336. 24954505 10.1038/onc.2014.173 PMC4275425

[33] Nagpal, J. K. , and B. R. Das . 2007 Identification of differentially expressed genes in tobacco chewing‐mediated oral cancer by differential display‐polymerase chain reaction. Eur. J. Clin. Invest. 37:658–664. 17635577 10.1111/j.1365-2362.2007.01841.x

[34] Beard, C. , K. Hochedlinger , K. Plath , A. Wutz , and R. Jaenisch . 2006 Efficient method to generate single‐copy transgenic mice by site‐specific integration in embryonic stem cells. Genesis 44:23–28. 16400644 10.1002/gene.20180

[35] Hochedlinger, K. , Y. Yamada , C. Beard , and R. Jaenisch . 2005 Ectopic expression of Oct‐4 blocks progenitor‐cell differentiation and causes dysplasia in epithelial tissues. Cell 121:465–477. 15882627 10.1016/j.cell.2005.02.018

[36] Tang, X. H. , B. Knudsen , D. Bemis , S. Tickoo , and L. J. Gudas . 2004 Oral cavity and esophageal carcinogenesis modeled in carcinogen‐treated mice. Clin. Cancer Res. 10:301–313. 14734483 10.1158/1078-0432.ccr-0999-3

[37] Wise‐Draper, T. M. , H. V. Allen , M. N. Thobe , E. E. Jones , K. B. Habash , K. Munger , et al. 2005 The human DEK proto‐oncogene is a senescence inhibitor and an upregulated target of high‐risk human papillomavirus E7. J. Virol. 79:14309–14317. 16254365 10.1128/JVI.79.22.14309-14317.2005 PMC1280217

[38] Ladang, A. , F. Rapino , L. C. Heukamp , L. Tharun , K. Shostak , D. Hermand , et al. 2015 Elp3 drives Wnt‐dependent tumor initiation and regeneration in the intestine. J. Exp. Med. 212:2057–2075. 26527802 10.1084/jem.20142288 PMC4647259

[39] Misuno, K. , X. Liu , S. Feng , and S. Hu . 2013 Quantitative proteomic analysis of sphere‐forming stem‐like oral cancer cells. Stem Cell Res. Ther. 4:156. 24423398 10.1186/scrt386 PMC4056689

[40] Chen, C. , B. Huang , M. Eliasson , P. Ryden , and A. S. Bystrom . 2011 Elongator complex influences telomeric gene silencing and DNA damage response by its role in wobble uridine tRNA modification. PLoS Genet. 7:e1002258. 21912530 10.1371/journal.pgen.1002258 PMC3164696

[41] Li, Q. , A. M. Fazly , H. Zhou , S. Huang , Z. Zhang , and B. Stillman . 2009 The elongator complex interacts with PCNA and modulates transcriptional silencing and sensitivity to DNA damage agents. PLoS Genet. 5:e1000684. 19834596 10.1371/journal.pgen.1000684 PMC2757915

[42] Moraitis, D. , B. Du , M. S. De Lorenzo , J. O. Boyle , B. B. Weksler , E. G. Cohen , et al. 2005 Levels of cyclooxygenase‐2 are increased in the oral mucosa of smokers: evidence for the role of epidermal growth factor receptor and its ligands. Cancer Res. 65:664–670. 15695412

[43] Adams, A. K. , G. E. Hallenbeck , K. A. Casper , Y. J. Patil , K. M. Wilson , R. J. Kimple , et al. 2015 DEK promotes HPV‐positive and ‐negative head and neck cancer cell proliferation. Oncogene 34:868–877. 24608431 10.1038/onc.2014.15 PMC4160430

[44] Wise‐Draper, T. M. , R. J. Morreale , T. A. Morris , R. A. Mintz‐Cole , E. E. Hoskins , S. J. Balsitis , et al. 2012 Future directions and treatment strategies for head and neck squamous cell carcinomas. Transl. Res. 160:167–177. 22683420 10.1016/j.trsl.2012.02.002 PMC3423575

[45] Wise‐Draper, T. M. , R. J. Morreale , T. A. Morris , et al. 2009 DEK proto‐oncogene expression interferes with the normal epithelial differentiation program. Am. J. Pathol. 174:71–81. 19036808 10.2353/ajpath.2009.080330 PMC2631320

[46] Rampias, T. , C. Sasaki , P. Weinberger , and A. Psyrri . 2009 E6 and e7 gene silencing and transformed phenotype of human papillomavirus 16‐positive oropharyngeal cancer cells. J. Natl Cancer Inst. 101:412–423. 19276448 10.1093/jnci/djp017

[47] Wu, Q. , Z. Li , H. Lin , L. Han , S. Liu , and Z. Lin . 2008 DEK overexpression in uterine cervical cancers. Pathol. Int. 58:378–382. 18477217 10.1111/j.1440-1827.2008.02239.x

[48] Tabbert, A. , F. Kappes , R. Knippers , J. Kellermann , F. Lottspeich , and E. Ferrando‐May . 2006 Hypophosphorylation of the architectural chromatin protein DEK in death‐receptor‐induced apoptosis revealed by the isotope coded protein label proteomic platform. Proteomics 6:5758–5772. 17001602 10.1002/pmic.200600197

[49] Mor‐Vaknin, N. , A. Punturieri , K. Sitwala , N. Faulkner , M. Legendre , M. S. Khodadoust , et al. 2006 The DEK nuclear autoantigen is a secreted chemotactic factor. Mol. Cell. Biol. 26:9484–9496. 17030615 10.1128/MCB.01030-06 PMC1698538

[50] Ko, S. I. , I. S. Lee , J. Y. Kim , S. M. Kim , D. W. Kim , K. S. Lee , et al. 2006 Regulation of histone acetyltransferase activity of p300 and PCAF by proto‐oncogene protein DEK. FEBS Lett. 580:3217–3222. 16696975 10.1016/j.febslet.2006.04.081

[51] Alexiadis, V. , T. Waldmann , J. Andersen , M. Mann , R. Knippers , and C. Gruss . 2000 The protein encoded by the proto‐oncogene DEK changes the topology of chromatin and reduces the efficiency of DNA replication in a chromatin‐specific manner. Genes Dev. 14:1308–1312. 10837023 PMC316669

[52] Scholzen, T. , and J. Gerdes . 2000 The Ki‐67 protein: from the known and the unknown. J. Cell. Physiol. 182:311–322. 10653597 10.1002/(SICI)1097-4652(200003)182:3<311::AID-JCP1>3.0.CO;2-9

[53] Gonzalez, M. A. , K. E. Tachibana , R. A. Laskey , and N. Coleman . 2005 Control of DNA replication and its potential clinical exploitation. Nat. Rev. Cancer 5:135–141. 15660109 10.1038/nrc1548

[54] Celis, J. E. , and A. Celis . 1985 Cell cycle‐dependent variations in the distribution of the nuclear protein cyclin proliferating cell nuclear antigen in cultured cells: subdivision of S phase. Proc. Natl Acad. Sci. USA 82:3262–3266. 2860667 10.1073/pnas.82.10.3262 PMC397755

