# Supplementary material for: Promotion of cell proliferation by the proto‐oncogene DEK enhances oral squamous cell carcinogenesis through field cancerization
Source: Cancer Med. 2017 Aug 23;6(10):2424–39. doi: 10.1002/cam4.1157 (PMC5633549; doi:10.1002/cam4.1157)
Supplement: Supplementary file 1 — Figure S1. Dek‐FLAG (exogenous) expression induced by DOX increased in three DOX‐inducible Dek‐FLAG KH2 ES clones. Figure S2. No difference between DOX+ and DOX‐ iDek mice in esophagus. Figure S3. No difference between DOX+ and DOX‐ iDek mice in skin and thymus after treatment with DOX(1 mg/L) for 2 weeks. Figure S4. The high frequency of esophagus tumors in DOX+ and DOX‐ iDek mice. Figure S5. DEK expression increased in the 4NQO‐ tongue carcinogenesis in both DOX+ and DOX‐iDek mice. Figure S6. Ki67 proliferation marker increased in the tongue carcinogenesis in both DOX+ and DOX‐ iDek mice, but showed no significant difference between DOX+ and DOX‐ iDek mice. Figure S7. No difference between p53 positive cells in tongue lesions of DOX+ and DOX‐ iDek mice. Figure S8. Western blot analyses for Sox9, PCNA, and Elp3 of tumor between overexpression of Dek or not. [file CAM4-6-2424-s001.pdf]

Supplemental Fig. 1

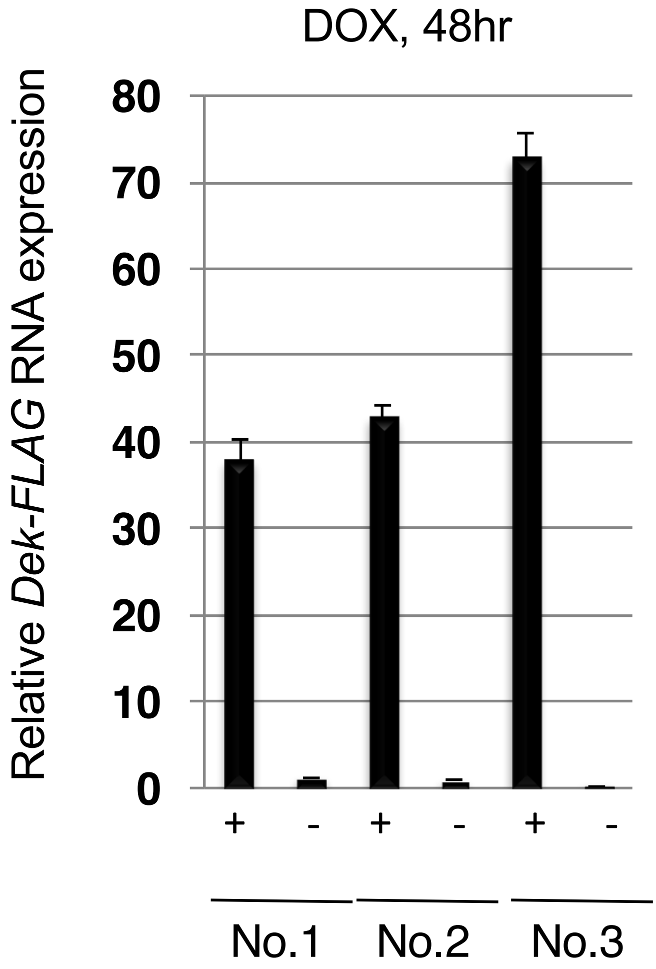

Supplementary Fig.1: *Dek-FLAG* (exogenous) expression induced by DOX increased in three DOX-inducible *Dek-FLAG* KH2 ES clones.

Relative *Dek-FLAG* RNA expression in three DOX-inducible *Dek-FLAG* KH2 ES clones. Data is averages ± SD.

Supplemental Fig. 2

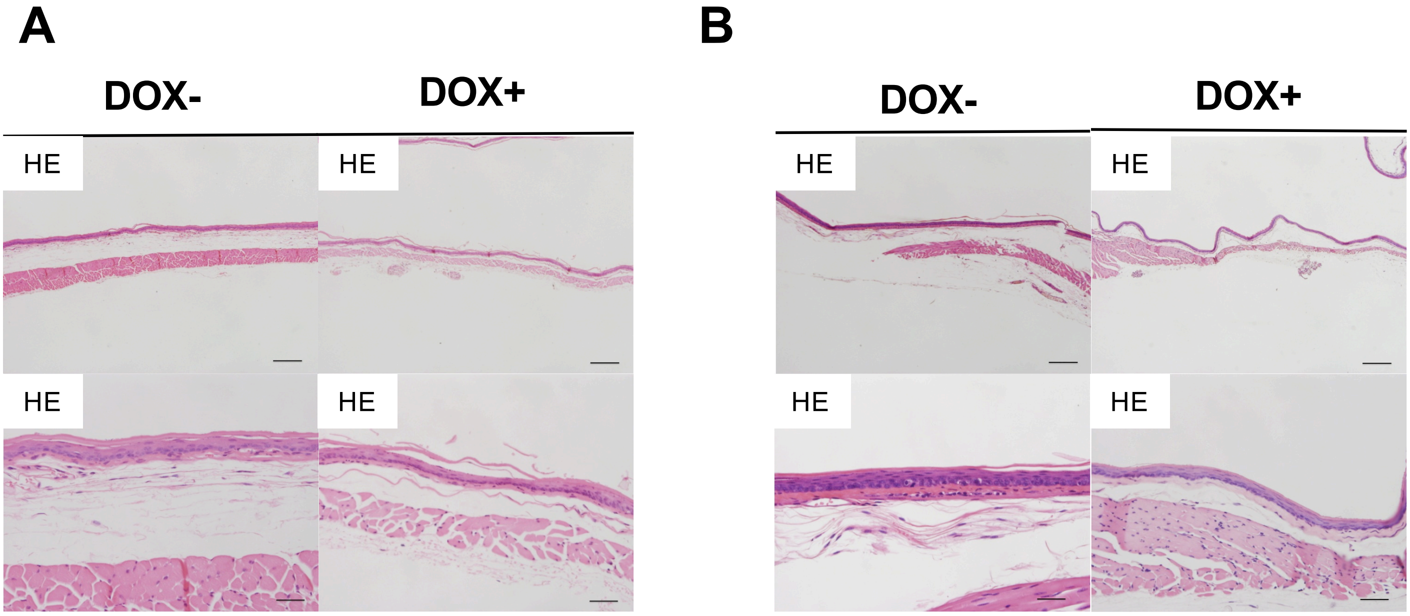

Supplemental Fig.2: No difference between DOX+ and DOX- *iDek* mice in esophagus.

(A) The HE sections in the esophagus of DOX+ and DOX- *iDek* mice (Rosa26-rtTA/TetO-*Dek* mice)
(B) The HE sections in the esophagus of DOX+ and DOX- *iDek-e* mice (Krt14-Cre/LSL-rtTA-ires-GFP/TetO-*Dek* mice)

Scale bars, 200μm at upper photos, 40μm at lower photos.

Supplementary Fig.S3

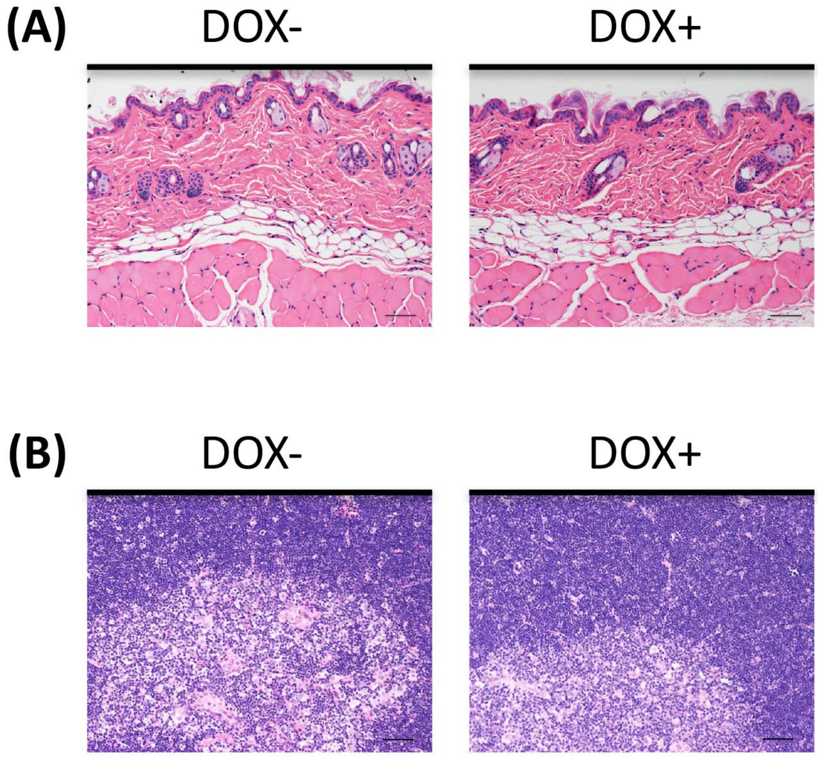

Supplementary Fig.S3: No difference between DOX+ and DOX- iDek mice in skin and thymus after treatment with DOX(1mg/L) for 2 weeks.
(A) The HE sections in the skin of DOX+ and DOX- *iDek* mice (Rosa26- rtTA/TetO-*Dek* mice) **(B)** The HE sections in the thymus of DOX+ and DOX- *iDek* mice (Rosa26- rtTA/TetO-*Dek* mice) *Scale bars*, 40μm at each photos.

Supplemental Fig. 4

A

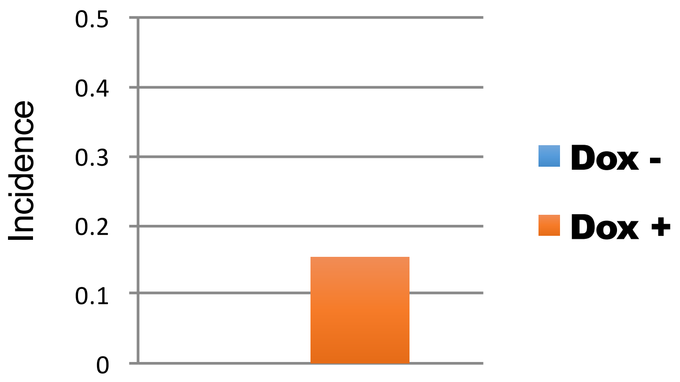

B

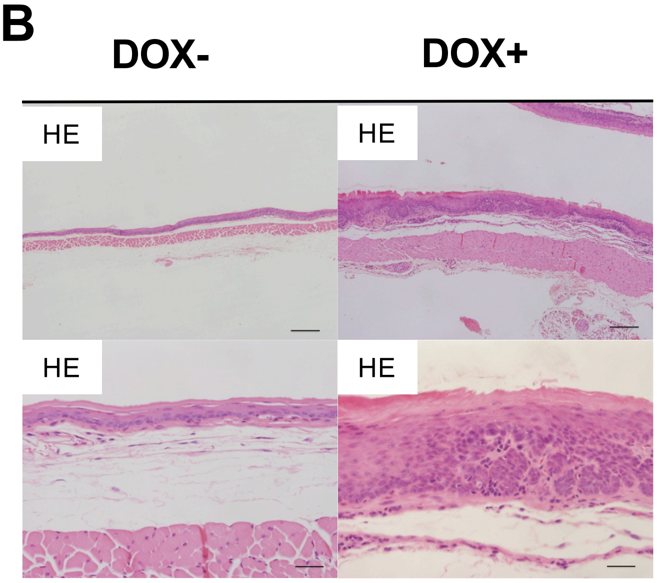

Supplemental Fig.3: The high frequency of esophagus tumors in DOX+ and DOX- *iDek mice*.

(A) Incidence of macroscopic tumors of the esophagus in DOX+ and DOX- *iDek* mice treated by 4NQO.

(B) The HE sections in the esophagus of DOX+ and DOX- mice. DOX+ *iDek* mice developed squamous cell carcinoma in the esophagus, but DOX- *iDek* mice did not.

Scale bars, 200μm at upper photos, 40μm at lower photos.

Supplemental Fig. 5

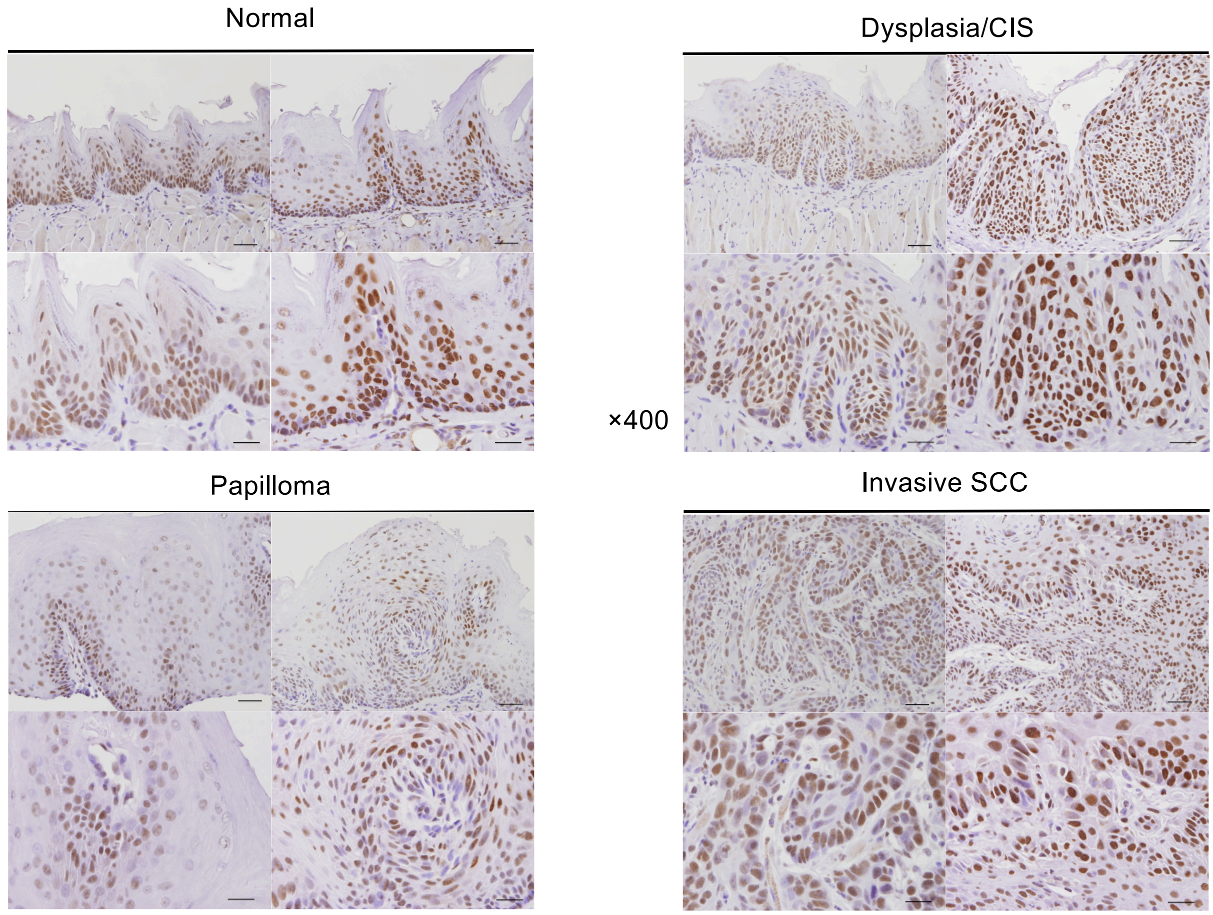

Supplemental Fig. 4: DEK expression increased in the 4NQO- tongue carcinogenesis in both DOX+ and DOX- *iDek* mice.

Representative photos of DEK staining in the tongue lesions of DOX- and DOX+ *iDek* mouse. *Scale bars*, 40μm.

Scale bars, 200μm at upper photos, 40μm at lower photos.

Supplemental Fig. 6

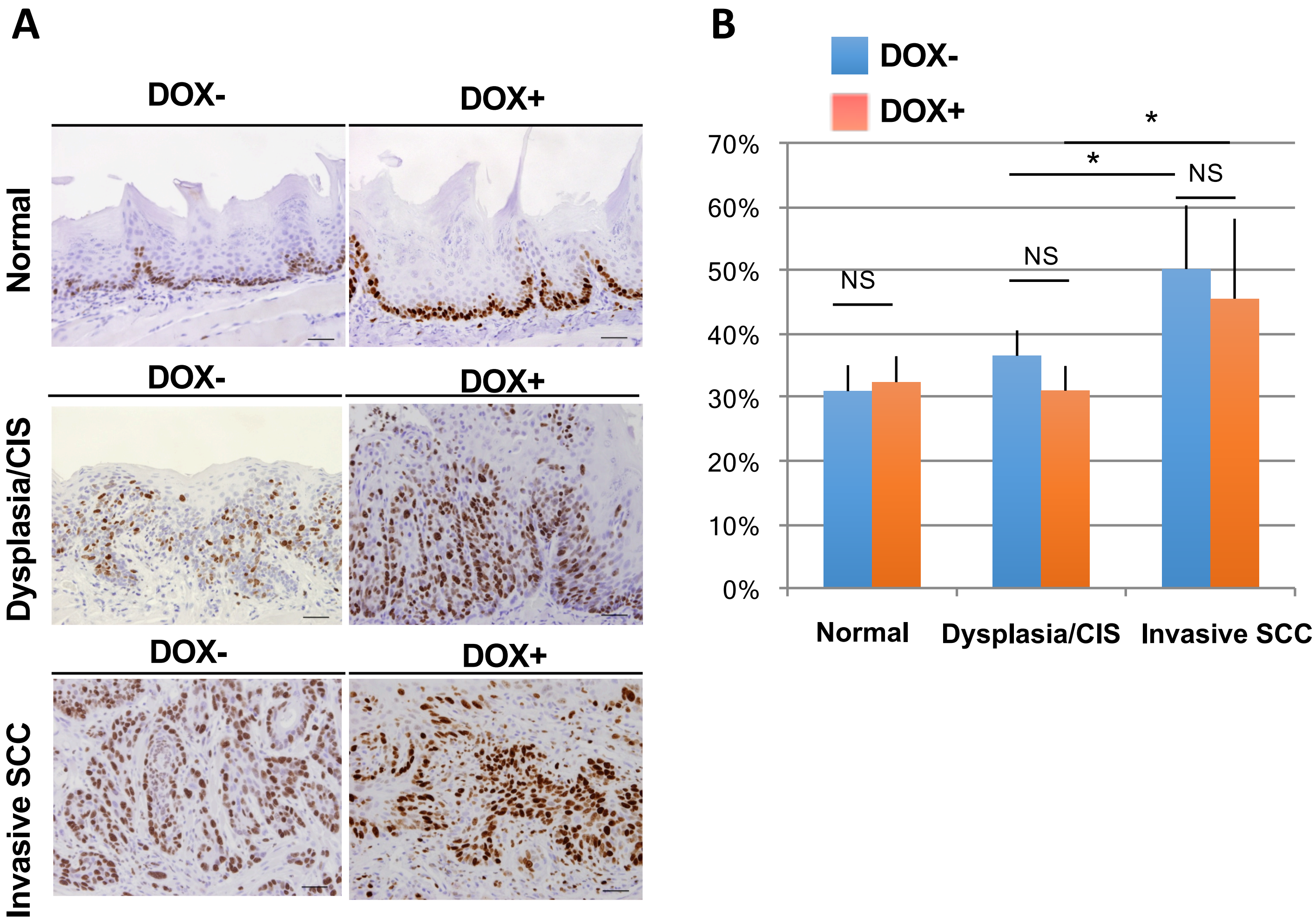

Supplemental Fig.5: Ki67 proliferation marker increased in the tongue carcinogenesis in both DOX+ and DOX- *iDek* mice, but showed no significant difference between DOX+ and DOX- *iDek* mice.

(A) Representative photos of Ki67 staining in the tongue lesions of DOX- and DOX+ *iDek* mouse. *Scale bars*, 40μm.
(B) The Ki67 positive index in tongue lesions of DOX+ and DOX- iDek mice treated by 4NQO. NS, Not Significant. Data are averages ± SD. **P<0.05. NS, Not significant.*

Supplementary Fig.S7

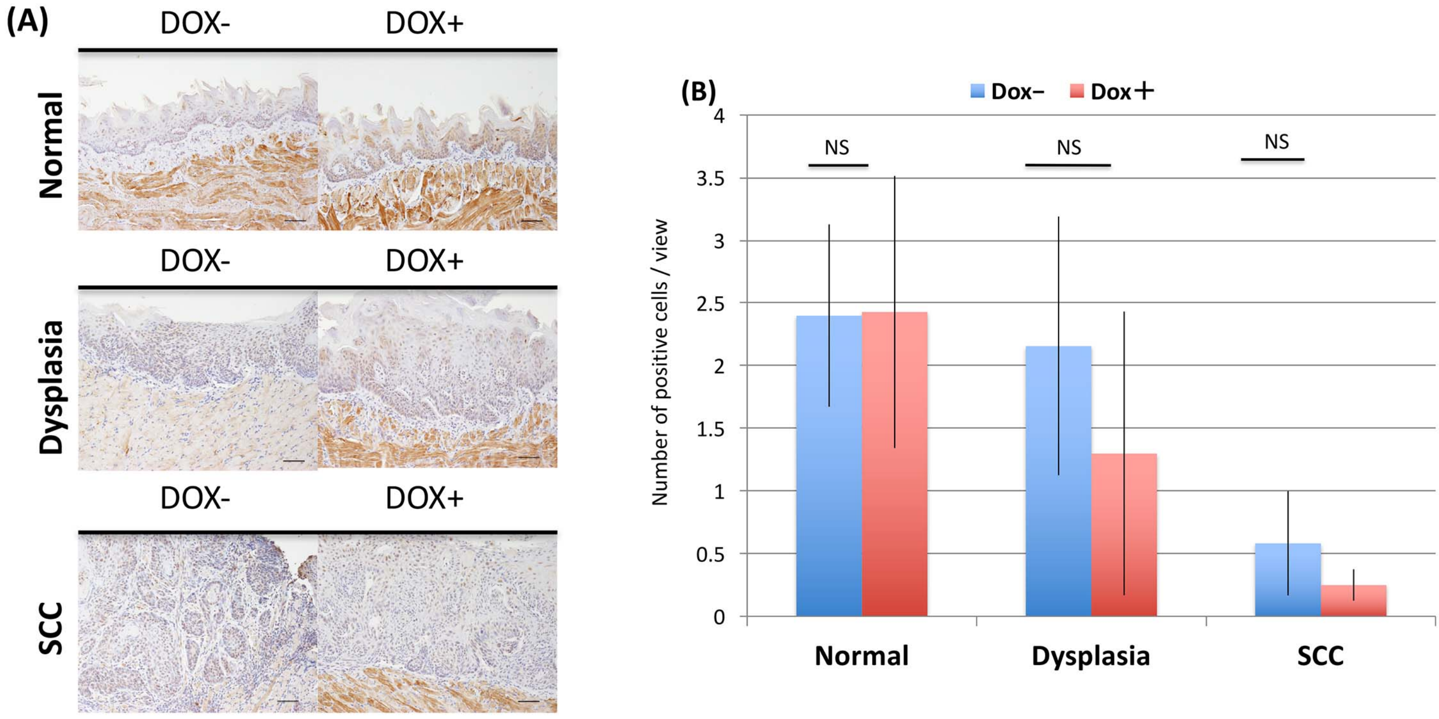

Supplementary Fig.S7: No difference between p53 positive cells in tongue lesions of DOX+ and DOX- iDek mice.
(A) Representative photos of P53 staining in the tongue lesions of DOX- and DOX+ *iDek* mouse. *Scale bars*, 40μm. **(B)** P53 positive cells in tongue lesions of DOX+ and DOX- iDek mice treated by 4NQO. Data are averages ±SD. **P<0.05.NS, Not significant.*

Supplementary Fig.S8

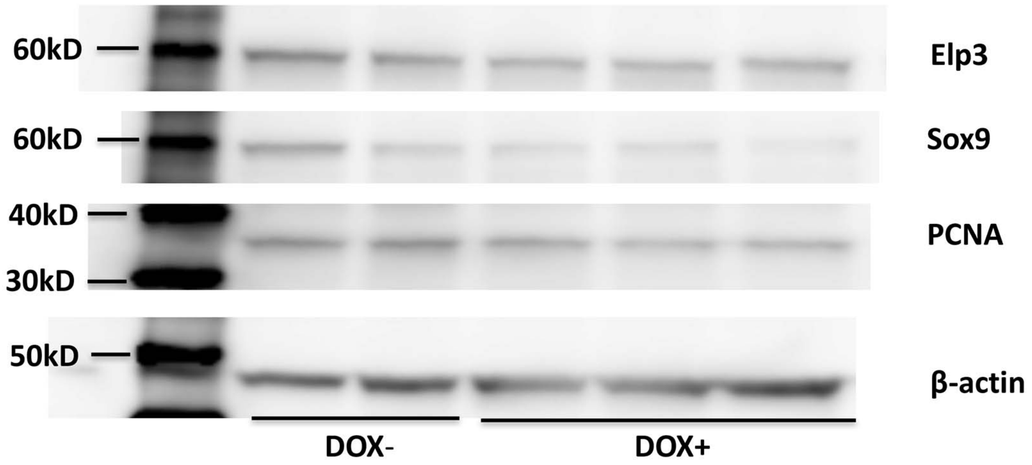

Supplementary Fig.S8: Western blot analyses for Sox9, PCNA, and Elp3 of tumor between overexpression of Dek or not .

Total protein extracts from tumor tissue sample of iDek mice treated with 4NQO and DOX (DOX+) or 4NQO only (DOX-). Indicated antibodies were used for Western blot analyses.
