# Supplementary material for: Promotion of cell proliferation by the proto‐oncogene DEK enhances oral squamous cell carcinogenesis through field cancerization
Source: Cancer Med. 2017 Aug 23;6(10):2424–39. doi: 10.1002/cam4.1157 (PMC5633549; doi:10.1002/cam4.1157)
Supplement: Supplementary file 2 — Table S1. PCR array data in the tongue tumors between DOX+ and DOX‐ iDek mice. [file CAM4-6-2424-s002.pdf]

Table S1

PCR Array Catalog #:	PAMM-178Z

Position	Unigene	Refseq	Symbol	Description	Gname	RT2 Catalog
A01	Mm.289914	NM_021421	Angel2	Angel homolog 2 (Drosophila)	2610307I21Rik/573	PPM37719A
A02	Mm.7214	NM_013470	Anxa3	Annexin A3	Anx3	PPM25413B
A03	Mm.1620	NM_009673	Anxa5	Annexin A5	Anx5/R74653	PPM04788A
A04	Mm.22478	NM_001080819	Arid1a	AT rich interactive domain 1A (SWI-like)	1110030E03Rik/BA	PPM26299C
A05	Mm.37623	NM_022989	Arl6ip6	ADP-ribosylation factor-like 6 interacting pr	2310057C01Rik/261	PPM28462A
A06	Mm.29133	NM_009773	Bub1b	Budding uninhibited by benzimidazoles 1 ho	AU045529/BUBR1	PPM27461A
A07	Mm.439957	NM_007572	C1qa	Complement component 1, q subcomponen	AI255395/C1q	PPM24525E
A08	Mm.2570	NM_009777	C1qb	Complement component 1, q subcomponen	-	PPM24831A
A09	Mm.439732	NM_007574	C1qc	Complement component 1, q subcomponen	AI385742/C1qg/Ciq	PPM24975B
A10	Mm.219527	NM_144938	C1s1	Complement component 1, s subcomponen	AA959438/AI25519	PPM41777B
A11	Mm.19131	NM_009778	C3	Complement component 3	AI255234/ASP/HSE	PPM04471E
A12	Mm.2408	NM_009779	C3ar1	Complement component 3a receptor 1	AZ3B/C3AR/HNFA	PPM04821A
B01	Mm.439678	NM_011413	C4a	Complement component 4A (Rodgers blood	Slp/SlpA	PPM68629A
B02	Mm.439678	NM_009780	C4b	Complement component 4B (Childo blood g	C4/Ss	PPM05780A
B03	Mm.247623	NM_007577	C5ar1	Complement component 5a receptor 1	C5aR/C5r1/Cd88/D	PPM34882A
B04	Mm.277665	NM_009788	Calb1	Calbindin 1	Brain-2/CB/Calb/C	PPM05029B
B05	Mm.1051	NM_009807	Casp1	Caspase 1	ICE/Il1bc	PPM02921E
B06	Mm.274927	NM_009912	Ccr1	Chemokine (C-C motif) receptor 1	Cmkbr1/Mip-1a-R	PPM03174A
B07	Mm.3460	NM_009841	Cd14	CD14 antigen	-	PPM06249G
B08	Mm.37426	NM_053094	Cd163	CD163 antigen	CD163v2/CD163v3	PPM06162B
B09	Mm.168789	NM_009876	Cdkn1c	Cyclin-dependent kinase inhibitor 1C (P57)	AL024410/CDKI/Ki	PPM02895B
B10	Mm.8655	NM_009888	Cfh	Complement component factor h	Mud-1/NOM/Sas-1	PPM25501F
B11	Mm.220362	NM_015780	Cfhr1	Complement factor H-related 1	AI194696/CFHRB/C	PPM33724A
B12	Mm.200608	NM_013492	Clu	Clusterin	AI893575/ApoJ/Cli/	PPM03567A
C01	Mm.103711	NM_009142	Cx3cl1	Chemokine (C-X3-C motif) ligand 1	AB030188/ABCD-3/	PPM02959F
C02	Mm.425692	NM_023158	Cxcl16	Chemokine (C-X-C motif) ligand 16	0910001K24Rik/AV	PPM03775A
C03	Mm.119162	NM_010485	Elavl1	ELAV (embryonic lethal, abnormal vision, D	2410055N02Rik/HU	PPM30921A
C04	Mm.29719	NM_028811	Elp3	Elongation protein 3 homolog (S. cerevisiae)	2610507P14Rik/KA	PPM27615A
C05	Mm.236645	NM_001043335	Eml1	Echinoderm microtubule associated protein	1110008N23Rik/A9	PPM34360A
C06	Mm.258397	NM_177821	Ep300	E1A binding protein p300	A430090G16/A7300	PPM05448E
C07	Mm.152289	NM_001164225	Fbxl16	F-box and leucine-rich repeat protein 16	BC042620/Scirr1	PPM31710A
C08	Mm.22673	NM_010185	Fcer1g	Fc receptor, IgE, high affinity I, gamma poly	AI573376/CD23/Fc	PPM04614F
C09	Mm.292276	NM_001122603	Fcgbp	Fc fragment of IgG binding protein	A430096B05Rik	PPM41225A
C10	Mm.150	NM_010186	Fcgr1	Fc receptor, IgG, high affinity I	AI323638/AV09295	PPM04014A
C11	Mm.425062	NM_010187	Fcgr2b	Fc receptor, IgG, low affinity IIb	AI528646/CD32/F6	PPM04611F
C12	Mm.22119	NM_010188	Fcgr3	Fc receptor, IgG, low affinity III	CD16	PPM04610G
D01	Mm.29891	NM_019739	Foxo1	Forkhead box O1	AI876417/Afxh/FKH	PPM03381C
D02	Mm.1239	NM_010277	Gfap	Glial fibrillary acidic protein	AI836096	PPM04716A
D03	Mm.467426	NM_008181	Gsta1	Glutathione S-transferase, alpha 1 (Ya)	Gst2-1/OTTMUSG0	PPM03858C
D04	Mm.347444	NM_008296	Hsf1	Heat shock factor 1	AA960185/Hsf1alph	PPM02939F
D05	Mm.334298	NM_028708	Jakmip3	Janus kinase and microtubule interacting pr	6330417G02Rik/AI5	PPM35844A
D06	Mm.243014	NM_019390	Lmna	Lamin A	Dhe	PPM34536A
D07	Mm.4105	NM_010721	Lmnb1	Lamin B1	-	PPM57673C
D08	Mm.7362	NM_010722	Lmnb2	Lamin B2	-	PPM25426F
D09	Mm.25642	NM_025520	Lsm5	LSM5 homolog, U6 small nuclear RNA asso	2010208O10Rik/231	PPM26793A
D10	Mm.282359	NM_008522	Ltf	Lactotransferrin	Csp82/Lf/MMS10R	PPM04406A
D11	Mm.252063	NM_010777	Mbp	Myelin basic protein	C76307/Hmbpr/R7	PPM04745F
D12	Mm.44174	NM_053164	Mrpl43	Mitochondrial ribosomal protein L43	4930442D21Rik/bM	PPM29032A
E01	Mm.30084	NM_019435	Ndufb11	NADH dehydrogenase (ubiquinone) 1 beta s	D5Bwg0566e/D5Bw	PPM27724A
E02	Mm.142253	NM_019482	Panx1	Pannexin 1	AI847747	PPM31514B
E03	Mm.24254	NM_011051	Pdcd6	Programmed cell death 6	AV299538/Alg2/MA	PPM26583A
E04	Mm.194486	NM_001081080	Phf3	PHD finger protein 3	2310061N19Rik/mK	PPM32771B
E05	Mm.39001	NM_172551	Polrmt	Polymerase (RNA) mitochondrial (DNA dire	1110018N15Rik/49	PPM28596A

		Fold Change (comparing to control group)		
		Group 1		
Position	Symbol	Fold Change	95% CI	Comments
A01	Angel2	1.9917	(1.51, 2.47)	OKAY
A02	Anxa3	0.7541	(0.47, 1.04)	OKAY
A03	Anxa5	0.9945	(0.78, 1.21)	OKAY
A04	Arid1a	0.9699	(0.00001, 2.28)	OKAY
A05	Arl6ip6	1.3263	(0.86, 1.79)	OKAY
A06	Bub1b	1.0757	(0.58, 1.57)	OKAY
A07	C1qa	0.2711	(0.00001, 1.60)	OKAY
A08	C1qb	5.4667	(1.70, 9.23)	OKAY
A09	C1qc	6.2794	(2.11, 10.45)	OKAY
A10	C1s1	1.7137	(0.01, 3.42)	OKAY
A11	C3	5.5778	(0.00001, 18.95)	OKAY
A12	C3ar1	2.7504	(0.00001, 8.13)	OKAY
B01	C4a	3.4931	(0.85, 6.14)	A
B02	C4b	112.3269	(0.00001, 399.13)	A
B03	C5ar1	3.7988	(0.00001, 8.50)	OKAY
B04	Calb1	5.6854	(0.00001, 24.25)	B
B05	Casp1	0.9298	(0.74, 1.11)	OKAY
B06	Ccr1	1.4652	(0.00001, 4.62)	OKAY
B07	Cd14	0.2167	(0.00001, 1.28)	OKAY
B08	Cd163	94.1558	(0.00001, 314.38)	A
B09	Cdkn1c	2.4094	(0.00001, 5.72)	OKAY
B10	Cfh	13.0993	(0.00001, 33.67)	OKAY
B11	Cfhr1	7.2628	(0.00001, 29.86)	B
B12	Clu	0.1999	(0.00001, 0.81)	OKAY
C01	Cx3cl1	0.7709	(0.29, 1.25)	OKAY
C02	Cxcl16	1.1593	(0.29, 2.03)	OKAY
C03	Elavl1	0.9282	(0.56, 1.29)	OKAY
C04	Elp3	1.4522	(1.20, 1.71)	OKAY
C05	Eml1	0.5995	(0.22, 0.98)	OKAY
C06	Ep300	0.0822	(0.00001, 0.48)	OKAY
C07	Fbxl16	2.5374	(0.00001, 5.74)	OKAY
C08	Fcer1g	1.675	(1.10, 2.25)	A
C09	Fcgbp	15.0611	(0.00001, 72.68)	A
C10	Fcgr1	14.1234	(0.00001, 40.22)	A
C11	Fcgr2b	4.4405	(0.00001, 10.75)	OKAY
C12	Fcgr3	0.8383	(0.00001, 4.63)	OKAY
D01	Foxo1	1.1202	(0.63, 1.61)	OKAY
D02	Gfap	0.9628	(0.00001, 2.65)	B
D03	Gsta1	1.2522	(0.16, 2.34)	OKAY
D04	Hsf1	1.1894	(0.95, 1.43)	OKAY
D05	Jakmip3	4.3829	(0.00001, 14.82)	B
D06	Lmna	0.7153	(0.30, 1.13)	OKAY
D07	Lmnb1	1.4435	(0.65, 2.23)	OKAY
D08	Lmnb2	2.1573	(0.00001, 4.38)	B
D09	Lsm5	2.2604	(0.85, 3.67)	OKAY
D10	Ltf	4.9457	(0.00001, 19.93)	OKAY
D11	Mbp	1.3166	(0.00001, 2.75)	OKAY
D12	Mrpl43	0.2069	(0.00001, 0.88)	OKAY
E01	Ndufb11	1.7865	(1.16, 2.41)	OKAY
E02	Panx1	1.6118	(0.85, 2.38)	OKAY
E03	Pdcd6	1.1093	(0.72, 1.50)	OKAY
E04	Phf3	1.4202	(0.93, 1.91)	OKAY
E05	Polrmt	0.967	(0.57, 1.36)	OKAY

E06	Mm.274589	NM_133931	Pot1a	Protection of telomeres 1A	1500031H18Rik/AI8	PPM36758A
E07	Mm.365961	NM_145541	Rap1a	RAS-related protein-1a	AI848598/G-22K/K	PPM05222A
E08	Mm.287609	NM_146042	Rnf144b	Ring finger protein 144B	BC025007/E130105	PPM37545A
E09	Mm.21567	NM_013650	S100a8	S100 calcium binding protein A8 (calgranuli	60B8Ag/AI323541/E	PPM05051F
E10	Mm.2128	NM_009114	S100a9	S100 calcium binding protein A9 (calgranuli	60B8Ag/AW546964,	PPM05050E
E11	Mm.229373	NM_001014761	Scn2b	Sodium channel, voltage-gated, type II, beta	2810451E09Rik/AI8	PPM41368B
E12	Mm.351459	NM_019812	Sirt1	Sirtuin 1 (silent mating type information reg	AA673258/SIR2L1/S	PPM05054A
F01	Mm.29760	NM_022433	Sirt3	Sirtuin 3 (silent mating type information reg	2310003L23Rik/AI8	PPM34583A
F02	Mm.25643	NM_181586	Sirt6	Sirtuin 6 (silent mating type information reg	2810449N18Rik/AI0	PPM26794A
F03	Mm.152699	NM_010754	Smad2	MAD homolog 2 (Drosophila)	7120426M23Rik/Ma	PPM04430C
F04	Mm.245715	NM_009222	Snap23	Synaptosomal-associated protein 23	23kDa/AA408749/S	PPM04251A
F05	Mm.4306	NM_009352	Terf1	Telomeric repeat binding factor 1	Pin2/Trbf1/Trf1	PPM04758A
F06	Mm.6402	NM_009353	Terf2	Telomeric repeat binding factor 2	TRF2	PPM05430E
F07	Mm.229292	NM_009360	Tfam	Transcription factor A, mitochondrial	AI661103/Hmgts/m	PPM39096B
F08	Mm.226554	NM_146074	Tfb1m	Transcription factor B1, mitochondrial	AI429207	PPM34025A
F09	Mm.390474	NM_008249	Tfb2m	Transcription factor B2, mitochondrial	Hkp1	PPM38181A
F10	Mm.29346	NM_145705	Tinf2	Terf1 (TRF1)-interacting nuclear factor 2	AW552114/D14Wsu	PPM37266A
F11	Mm.87596	NM_011905	Tlr2	Toll-like receptor 2	Ly105	PPM04220B
F12	Mm.38049	NM_021297	Tlr4	Toll-like receptor 4	Lps/Ly87/Ran/M1/	PPM04207F
G01	Mm.208477	NM_028343	Tmem135	Transmembrane protein 135	2810439K08Rik/AW	PPM33200A
G02	Mm.23217	NM_028975	Tmem33	Transmembrane protein 33	1110006G02Rik/160	PPM26408A
G03	Mm.103551	NM_023764	Tollip	Toll interacting protein	4930403G24Rik/493	PPM06269E
G04	Mm.20837	NM_009906	Tpp1	Tripeptidyl peptidase I	Cln2/TPP-I	PPM26098C
G05	Mm.410189	NM_023719	Txnip	Thioredoxin interacting protein	1200008J08Rik/AA6	PPM05656C
G06	Mm.241454	NM_177184	Vps13c	Vacuolar protein sorting 13C (yeast)	AI552542/AV02604	PPM32619A
G07	Mm.290950	NM_172767	Vwa5a	Von Willebrand factor A domain containing	5830475I06Rik/AW5	PPM37846A
G08	Mm.228805	NM_011721	Wrn	Werner syndrome homolog (human)	AI846146	PPM05058C
G09	Mm.103262	NM_177660	Zbtb10	Zinc finger and BTB domain containing 10	4832414A18/AU021	PPM30607A
G10	Mm.106970	NM_011763	Zfp9	Zinc finger protein 9	1810048F22Rik/AU	PPM30728A
G11	Mm.273496	NM_011767	Zfr	Zinc finger RNA binding protein	C920030H05Rik	PPM36666A
G12	Mm.34399	NM_172700	Zmpste24	Zinc metallopeptidase, STE24 homolog (S. c	A530043O15Rik/D0	PPM28203A
H01	Mm.328431	NM_007393	Actb	Actin, beta	Actx/E430023M04R	PPM02945B
H02	Mm.163	NM_009735	B2m	Beta-2 microglobulin	Ly-m11/beta2-m/b	PPM03562A
H03	Mm.309092	NM_008084	Gapdh	Glyceraldehyde-3-phosphate dehydrogenas	Gapd	PPM02946E
H04	Mm.3317	NM_010368	Gusb	Glucuronidase, beta	AI747421/Gur/Gus/	PPM05490C
H05	Mm.2180	NM_008302	Hsp90ab1	Heat shock protein 90 alpha (cytosolic), clas	90kDa/AL022974/C	PPM04803F
H06	N/A	SA_00106	MGDC	Mouse Genomic DNA Contamination	MIGX1B	
H07	N/A	SA_00104	RTC	Reverse Transcription Control	RTC	PPX63340A
H08	N/A	SA_00104	RTC	Reverse Transcription Control	RTC	PPX63340A
H09	N/A	SA_00104	RTC	Reverse Transcription Control	RTC	PPX63340A
H10	N/A	SA_00103	PPC	Positive PCR Control	PPC	
H11	N/A	SA_00103	PPC	Positive PCR Control	PPC	
H12	N/A	SA_00103	PPC	Positive PCR Control	PPC	

E06	Pot1a	0.127	(0.00001, 0.66)	OKAY
E07	Rap1a	1.928	(1.09, 2.77)	OKAY
E08	Rnf144b	1.7739	(0.86, 2.69)	OKAY
E09	S100a8	4.246	(0.00001, 13.21)	OKAY
E10	S100a9	2.8736	(0.00001, 9.90)	OKAY
E11	Scn2b	11.7429	(0.00001, 54.11)	B
E12	Sirt1	0.1744	(0.00001, 0.98)	OKAY
F01	Sirt3	1.5708	(0.92, 2.22)	OKAY
F02	Sirt6	0.9151	(0.51, 1.32)	OKAY
F03	Smad2	1.314	(0.87, 1.75)	OKAY
F04	Snap23	1.0494	(0.53, 1.57)	OKAY
F05	Terf1	0.5061	(0.00001, 1.46)	OKAY
F06	Terf2	0.7053	(0.00001, 1.55)	OKAY
F07	Tfam	1.626	(1.29, 1.96)	OKAY
F08	Tfb1m	1.3328	(0.20, 2.47)	OKAY
F09	Tfb2m	1.6979	(1.27, 2.13)	OKAY
F10	Tinf2	1.2934	(0.83, 1.76)	OKAY
F11	Tlr2	1.6067	(0.59, 2.62)	OKAY
F12	Tlr4	0.1402	(0.00001, 0.79)	OKAY
G01	Tmem135	1.6377	(0.80, 2.47)	OKAY
G02	Tmem33	0.7978	(0.16, 1.43)	OKAY
G03	Tollip	1.3141	(0.93, 1.70)	OKAY
G04	Tpp1	0.9645	(0.47, 1.46)	OKAY
G05	Txnip	1.9411	(0.00001, 6.56)	OKAY
G06	Vps13c	0.2229	(0.00001, 1.19)	OKAY
G07	Vwa5a	2.5065	(0.72, 4.29)	OKAY
G08	Wrn	1.4362	(0.93, 1.94)	OKAY
G09	Zbtb10	4.2968	(0.00001, 10.31)	OKAY
G10	Zfp9	1.8668	(0.17, 3.56)	OKAY
G11	Zfr	0.0794	(0.00001, 0.50)	OKAY
G12	Zmpste24	0.128	(0.00001, 0.70)	OKAY
H01	Actb	0.846	(0.48, 1.21)	OKAY
H02	B2m	3.2628	(0.39, 6.14)	OKAY
H03	Gapdh	0.929	(0.45, 1.41)	OKAY
H04	Gusb	1.4818	(0.88, 2.08)	OKAY
H05	Hsp90ab1	0.3899	(0.00001, 1.06)	OKAY
H06	MGDC	1.7309	(0.00001, 6.09)	C
H07	RTC	0.924	(0.20, 1.64)	OKAY
H08	RTC	0.9141	(0.21, 1.62)	OKAY
H09	RTC	0.924	(0.21, 1.64)	OKAY
H10	PPC	0.9355	(0.43, 1.44)	OKAY
H11	PPC	0.1389	(0.00001, 0.66)	OKAY
H12	PPC	0.0341	(0.00001, 0.28)	OKAY
