# Supplementary material for: Promotion of cell proliferation by the proto‐oncogene DEK enhances oral squamous cell carcinogenesis through field cancerization
Source: Cancer Med. 2017 Aug 23;6(10):2424–39. doi: 10.1002/cam4.1157 (PMC5633549; doi:10.1002/cam4.1157)
Supplement: Supplementary file 3 — Table S2. Primers used for quantitative real‐time RT‐PCR. [file CAM4-6-2424-s003.pdf]

Table S2. Primers used for quantitative real-time PCR.

Target gene	Primer sequences (5'-3')	PCR products (bp)
Elp3	F:CAGTCCCTCCTCACTATCGAA<br>R:TCTGTGGGGTTTGCACATCAC	107
Sox9	F:AGTACCCGCATCTGCACAAC<br>R:ACGAAGGGTCTCTTCTCGCT	88
Pcna	F:TTGCACGTATATGCCGAGACC<br>R:GGTGAACAGGCTCATTCATCTCT	183
Ptgs2	F:TTCCAATCCATGTCAAAACCGT<br>R:AGTCCGGGTACAGTCACACTT	76
Mcm2	F:ATCCACCACCGCTTCAAGAAC<br>R:TACCACCAAACTCTCACGGTT	114
Cdc6	F:GACACAAGCTACCATCGGTTT<br>R:CAGGCTGGACGTTTCTAAGTTT	101
Mcm3	F:AGCGCAGAGAGACTACTTGGA<br>R:GCGGTTAGCCCTCTTTTCATTC	151
Mcm4	F:TCTTTGACCGTTATCCTGACTCC<br>R:TGCCTCGATCTATCTCCACCC	228
