# Supplementary material for: Promotion of cell proliferation by the proto‐oncogene DEK enhances oral squamous cell carcinogenesis through field cancerization
Source: Cancer Med. 2017 Aug 23;6(10):2424–39. doi: 10.1002/cam4.1157 (PMC5633549; doi:10.1002/cam4.1157)
Supplement: Supplementary file 4 — Data S1. Details of experimental methods in this study. [file CAM4-6-2424-s004.docx]

**Supplementary Materials and Methods**

**Promotion of cell proliferation by the proto-oncogene *DEK* enhances oral squamous cell carcinogenesis through field cancerization**

Takayuki Nakashima et al.

***Generation of Dek-inducible mouse embryonic stem (ES) cell lines***

*Dek*-inducible ES cell lines were generated using a modification of a previously described protocol ^34^. Mouse *Dek* cDNA was amplified by polymerase chain reaction (PCR) from mouse ES cells, ligated into a 3X*FLAG* sequence (Sigma, St. Louis, MO, USA), and then cloned into a flip-in vector consisting of a frt-flanked neomycin-selectable marker and a promoterless, ATG-less hygromycin-resistance gene downstream from a tet-operon. The *Dek*-flip-in vector was electroporated with 50 μg of *Dek-*flip-in vector and 25 μg of pCAAGS-FLPe-puro into 1 × 10^7^ KH2 ES cells at 500 V and 25 μF using a Gene PulserII (Bio-Rad, Hercules, CA, USA). KH2 ES cells permitted the expression of the M2-rtTA tetracycline-responsive transactivator under the control of the ROSA26 promoter.

***Generation of Dek-inducible mice***

The *Dek*-inducible ES cell clone was injected into ICR mouse embryos at the 8-cell stage to generate chimeras at Osaka University. These chimeras yielded germ line transmission and were crossed with C57BL/6 mice. Finally, we obtained *Rosa26-M2rtTA* and *Tet-O-Dek* mice.

***Doxycycline (DOX) treatment***

To induce DEK expression in vitro, DOX was diluted in ES cell culture medium to a final concentration of 1 mg/mL. In murine experiment, DOX was added to drinking water at a final concentration of 2 mg/mL.

***4-Nitroquinoline 1-oxide (4NQO) carcinogen exposure***

Mice were treated with 4NQO for induction of malignant lesions. Mice were fed a basal diet (CE-2; CLEA Japan, Tokyo, Japan) until termination of the study. Genotypes were identified by PCR analysis of their tail DNA using allele-specific primers. Treatment with 4NQO was carried out by addition of 4NQO to the drinking water at a concentration of 20 ppm.

***Preparation of tissue samples for counting and histological analysis***

The tongue and esophagus were removed from mice, and the esophagus was opened along the longitudinal axis. The number and longest diameter of the tumors in the tongue and esophagus were measured using a dissecting microscope. To eliminate interobserver error, all counts were performed by a single observer who was blinded to the genotypes of the mice. All of the samples were also evaluated by a second observer to confirm the results of the first observer. After tumor counting, the tongues were cut at the longitudinal center and were processed for assessment of microscopic lesions. The incidences and multiplicities of microscopic lesions in the tongue and esophagus were determined in histological sections at the longitudinal center of the tongue and esophagus. All of the excised tissues were fixed for 24 h in neutral-buffered 10% formalin. The fixed samples were processed by standard methods, embedded in paraffin, sectioned at 3-μm thickness, and stained with hematoxylin and eosin (HE) staining.

***Immunohistochemistry (IHC)***

IHC analysis of human tissues was performed using a Vectastain ABC kit (Vector Laboratories, Burlingame, CA, USA). Tissue sections were cut to a thickness of 3 μm, deparaffinized, placed in 50 mM citrate buffer (pH 6.0), and heated in an autoclave at 120°C for 1 min. Endogenous peroxidase activity was blocked by incubation for 10 min in 5% H_2_O_2_ in methanol at room temperature. After washing three times with PBS, tissue sections were pre-incubated with normal blocking serum for 40 min at room temperature and then incubated with anti-DEK (human: 1:200 dilution [BD, CA, USA]; mouse: 1:400 dilution [Proteintech, IL, USA]), anti-Ki67 (human: 1:200 dilution [Dako, CA, USA]; mouse: 1:400 dilution [Abcam, Cambridge, UK]), anti-PCNA (1:200 dilution; Dako), anti-FLAG (1:500 dilution; Sigma), anti-p53 (rabbit 1:400 dilution [Sigma-Aldrich]) overnight at 4°C. Subsequently, the tissue sections were incubated with biotinylated secondary antibody at room temperature for 30 min and then incubated with ABC reagent according to the manufacturer’s instructions. The sections were developed with 3,3’-diaminobenzidine (DAB) and then counterstained with hematoxylin. No specific staining was observed in the negative control slides prepared without primary antibody and with suitable rodent IgG.

For immunofluorescent staining, after treatment with primary antibodies, samples were incubated for 30 min at room temperature with FITC- or rhodamine-conjugated secondary antibodies (1:500 dilution; Dako). DNA was labeled with 4′,6-diamidino-2-phenylindole.

***Evaluation (index) of immunohistochemical staining***

All specimens were examined by two pathologists who did not possess knowledge of the clinical data. When discrepancies occurred, a final score was established by reassessment on a double-headed microscope. All the samples were assessed using five fields at a magnification of 200×. The average of five views was defined as the index of the samples.

***Real-time RT-PCR***

Total RNA was extracted using an RNeasy kit (Qiagen, TX, USA) according to the manufacturer’s protocol. cDNAs were synthesized using a SuperScript III First-Strand Synthesis Kit (Life Technologies, Inc., MD, USA). Quantitative real-time RT-PCR was performed using a StepOnePlus system (Applied Biosystems, Foster City, CA, USA). The PCR primers are listed in **Supplementary Table S2**. To analyze relative gene expression data, we used the comparative Ct method. Two independent experiments were performed, with duplicate reactions in each experiment. Beta-actin was used for the normalization as a housekeeping gene.

***PCR array analysis***

The expression levels of 84 ageing genes were determined using the mouse ageing RT2 Profiler PCR array (Qiagen, Frederick, MD, USA). Total RNA was extracted from tumour samples using an RNeasy Mini Kit (Qiagen, TX, USA). cDNA was synthesized from 0.3 μg total RNA using an RT2 PCR Array First Strand kit (Qiagen). Real-time PCR was performed in 96-well plates using RT2 SYBR Green qPCR Master Mix (Qiagen) and the StepOnePlus Real-Time PCR System (Applied Biosystems, Foster City, CA, USA) in triplicate. All the genes and data are listed in **Supplementary Table S1**. The results were normalised using five housekeeping genes and analysed by the comparative Ct method.

***Western blot analysis***

Total protein was extracted with RIPA buffer (Thermo 89900) from tumor tissue. Tumor tissues were obtained from tongue of iDek mice witch were treated with 4nqo and DOX+ or 4nqo only. Equal amounts of protein (75 μg) were separated on 10% SDS-PAGE and blotted onto PVDF membranes (Millipore). For detection of Sox9, we used Ab26414 (abcam), and for detection of Elp3, we used Ab113228 (Abcam), and for detection of PCNA, we used Sc-9857 (Santa cruz), and for β-actin, we used 4967s (Cell signaling). For secondary antibodies, we used horseradish peroxidase (HRP)–linked anti-rabbit or HRP–linked anti-goat (DAKO), both at a 1:2,000 dilution. Detection was conducted by chemiluminescence using enhanced chemiluminescence.

***Microarray analysis***

For microarray analysis, total RNA was extracted using a Simply RNA Tissue Kit (Promega, Fitchburg, WI, USA) on a Maxwell RSC instrument. Gene expression analysis of the RNA samples was performed by Takara Bio Inc. (Shiga, Japan) using an Agilent Expression Array (SurePrint G3 Mouse GE 8 × 60 K Microarray). The obtained data were analysed and visualised with MeV MultiExperiment Viewer and DAVID. Genes that were differentially expressed between DOX+ and DOX- *iDek* mice were identified based on a fold-change of at least 2 (upregulated) or of less than 0.5 (downregulated) and a *p* value of less than 0.01. All microarray data were deposited in the Gene Expression Omnibus (GEO) under dataset accession no. GSE87587 (http://www.ncbi.nlm.nih.gov/geo/).

***Statistical analysis***

For comparisons between the two groups, the data were analysed using Student’s t-tests, Mann-Whitney U tests, and Fisher’s extract tests. Differences with *P* values less than 0.05 were considered statistically significant.
